# Inhibition of TWEAK/Tnfrsf12a axis protects against acute liver failure by suppressing RIPK1-dependent apoptosis

**DOI:** 10.1038/s41420-022-01123-0

**Published:** 2022-07-19

**Authors:** Zhijie Li, Heming Wang, Junjin Zhu, Ning Nan, Yi Lin, Xuran Zhuang, Ling Li, Yamin Zhang, Pengyu Huang

**Affiliations:** 1 School of Life Science and Technology, ShanghaiTech University, Shanghai, 201210 China; 2 University of Chinese Academy of Sciences, Beijing, 100049 China; 3 CAS Center for Excellence in Molecular Cell Science, Chinese Academy of Sciences, Shanghai, 200031 China; 4 Department of Gastroenterology and Hepatology, Zhongshan Hospital, Fudan University, Shanghai, 200032 China; 5 Tianjin First Central Hospital, Tianjin, 300190 China; 6 Institute of Biomedical Engineering, Chinese Academy of Medical Sciences and Peking Union Medical College, Tianjin, 300192 China

**Keywords:** Apoptosis, Physiology

## Abstract

Acute liver failure (ALF) is a severe clinical syndrome characterized by massive death of hepatocytes in a short time, resulting in coagulopathy and hepatic encephalopathy, with a high mortality in patients without pre-existing liver disease. Effective treatment of ALF is currently limited to liver transplantation, highlighting the need for new target therapies. Here, we found that expression of hepatic tumor necrosis factor-like weak inducer of apoptosis (TWEAK) and its receptor tumor necrosis factor receptor superfamily member 12A (Tnfrsf12a) were significantly increased during ALF induced by thioacetamide (TAA) or acetaminophen (APAP). Inhibition of TWEAK/Tnfrsf12a axis markedly attenuated TAA or APAP-induced ALF. Moreover, our results demonstrated that TWEAK/Tnfrsf12a axis induced receptor-interacting protein kinase 1 (RIPK1)-dependent apoptosis of hepatocytes, instead of necroptosis or pyroptosis. Notably, hepatic TNFRSF12A and TWEAK levels were also significantly increased in liver biopsies from ALF patients. In summary, our results demonstrate that during ALF, TWEAK/Tnfrsf12a axis activates RIPK1 in hepatocytes, leading to RIPK1-dependent apoptosis and subsequent liver injury. Therefore, inhibition of either TWEAK/Tnfrsf12a axis or RIPK1-dependent apoptosis attenuates liver injury, providing a new potential therapeutic target for the treatment of ALF.

## Introduction

Acute liver failure (ALF) is characterized by massive necrosis of hepatocytes, resulting in severe liver dysfunction and sometimes death within hours or days [1]. ALF can be induced by an acute viral infection, drugs, or autoimmune hepatitis. Of these, acetaminophen (APAP) overdose is the leading cause of ALF in many developed countries because of its extensive use as an analgesic [2]. Currently, the molecular basis underlying ALF is largely unknown, and therapeutic options are very limited due to the lack of effective therapeutic targets. Thus, liver transplantation is often the only choice for patients with end-stage ALF. However, liver donors are in short supply, raising an urgent need for novel therapeutic approaches for ALF.

Here, we found that the expression of hepatic tumor necrosis factor-like weak inducer of apoptosis (TWEAK) and its receptor tumor necrosis factor receptor superfamily member 12A (Tnfrsf12a) significantly increased during thioacetamide (TAA) or APAP-induced ALF. TWEAK is a TNF superfamily cytokine that can activate the Tnfrsf12a receptor [3, 4]. Persistent TWEAK-Tnfrsf12a engagement or Tnfrsf12a overexpression contributes to acute ischemic stroke, rheumatoid arthritis, systemic lupus erythematosus, multiple sclerosis, and cancer [5, 6]. Recently, TWEAK/Tnfrsf12a axis was also reported to regulate necroptosis and contribute to acute kidney injury [7, 8]. However, the role of TWEAK/Tnfrsf12a axis during ALF remains unknown.

In this study, we found that inhibition of TWEAK/Tnfrsf12a axis significantly attenuated ALF induced by TAA or APAP. We additionally showed that TWEAK/Tnfrsf12a axis activated RIPK1, inducing RIPK1-dependent apoptosis of hepatocytes in vitro. Moreover, ALF induced by TAA or APAP could also be attenuated by either RIPK1 or pan-caspase inhibitors, and the role of necroptosis and pyroptosis were excluded using genetic approaches. Thus, hepatocytes undergo RIPK1-dependent apoptosis during ALF.

Notably, upregulation of hepatic TWEAK and TNFRSF12A levels was also observed in liver samples from ALF patients, suggesting a potential role for TWEAK/TNFRSF12A axis in human ALF patients. In summary, we demonstrate that ALF is driven by TWEAK/Tnfrsf12a axis through RIPK1-dependent apoptosis, providing a new therapeutic target for the treatment of ALF.

## Results

### Transcriptional changes in the livers of ALF mice

**Fig. 1:**
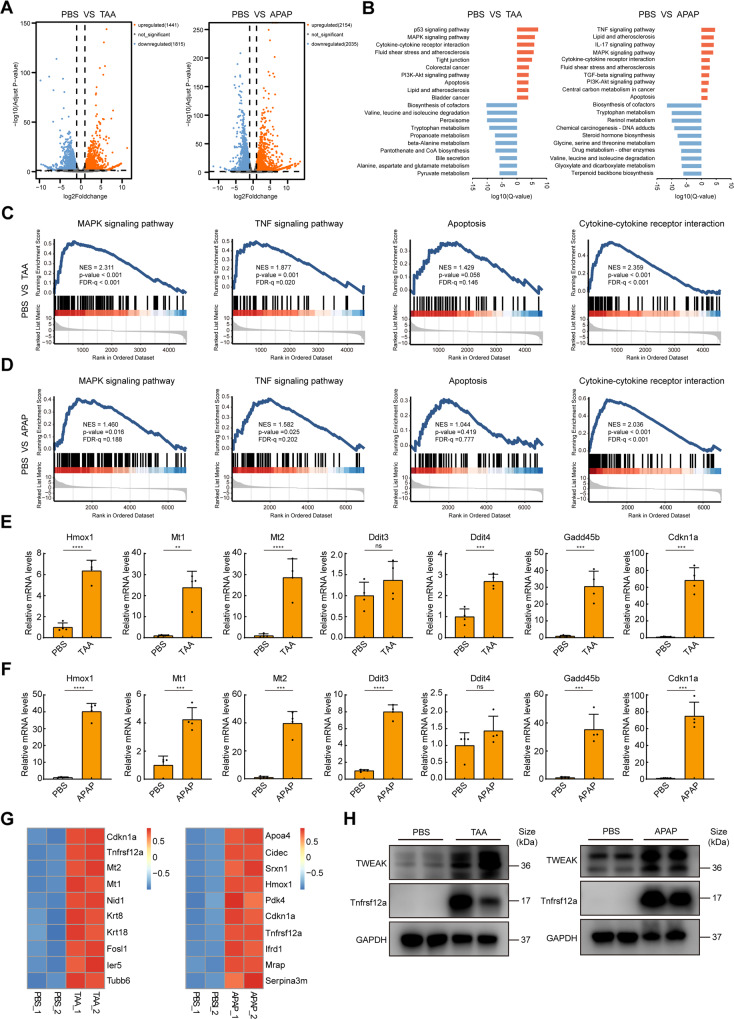
Transcriptome analysis of livers in murine ALF models. **A** Volcano plot indicating DEGs between normal mice and ALF mice, *n* = 2 for each group. **B** KEGG pathway analysis of DEGs showing the top pathways that are upregulated or downregulated during ALF. **C**, **D** GSEA analysis of MAPK, TNF, apoptosis, and cytokine-cytokine receptor interaction signaling pathways. **E**, **F** qPCR analysis of indicated mRNA levels in livers of PBS-treated mice and ALF mice (*n* = 4; mean ± s.d.). *P* values were determined by Student’s *t* test (**P < 0.01; ***P < 0.001; ****P < 0.0001; ns, not significant). **G** Heatmap showing top list out of commonly upregulated genes between TAA or APAP-induced ALF. **H** Expression of TWEAK and Tnfrsf12a of whole liver lysates from PBS-treated controls and ALF mice, *n* = 2 for each group. GAPDH was used for normalization.

To explore the potential molecular basis of ALF and identify new therapeutic targets, we performed a full transcriptome sequencing analysis of liver samples from ALF mice induced by TAA or APAP toxicity and PBS-treated controls (Figs. 1A and S1A). Pathway analysis (KEGG) identified the top upregulated and downregulated signaling pathways during ALF (Fig. 1B). Of these, downregulated signaling pathways were mostly related to metabolism. However, apoptosis and inflammatory signaling pathways were upregulated in the ALF group. Gene set enrichment analysis (GSEA) revealed a relatively high consistency with the KEGG results (Fig. 1C, D).

Considering that cell death and inflammation are the leading cause of ALF, we next analyzed differentially expressed genes (DEGs) that were commonly upregulated in these murine ALF models. DEGs analysis identified 660 commonly upregulated genes between TAA and APAP group (Fig. S1B). Among these genes, expression of heme oxygenase 1 (Hmox1), metallothionein 1 (Mt1), and metallothionein 2 (Mt2) were significantly upregulated, suggesting acute oxidative stress response during ALF (Fig. 1E, F). Moreover, the expression of genes related to DNA damage and cell cycle arrest (such as DNA-damage-inducible transcript 3 (Ddit3), DNA-damage-inducible transcript 4 (Ddit4), growth arrest and DNA-damage-inducible 45 beta (Gadd45b) and cyclin-dependent kinase inhibitor 1A (Cdkn1a)) were increased (Fig. 1E, F). In addition, a variety of cytokines and chemokines (tumor necrosis factor α (TNF-α), interleukin 6 (IL-6), interleukin 1 beta (IL-1β), chemokine (C-C motif) ligand 2 (Ccl2) and chemokine (C-X-C motif) ligand 2 (Cxcl2)) were also elevated in ALF groups, indicating increased immune cell infiltration and intense immune response (Fig. S1C–F).

Moreover, the top list of commonly upregulated genes in these ALF models were shown in the heatmap (Fig. 1G). Besides Cdkn1a mentioned above, we also found TNF superfamily receptor Tnfrsf12a was among these. Tnfrsf12a is TNF receptor family member that is activated by the cytokine TWEAK, a typical member of the TNF ligand family [3, 4]. Recently, TWEAK/Tnfrsf12a axis was reported to regulate necroptosis and contribute to acute kidney injury [7, 8]. However, the role of TWEAK/Tnfrsf12a axis during ALF remains unknown. Therefore, we next assessed the involvement of TWEAK/Tnfrsf12a axis during ALF. The expression of hepatic TWEAK and Tnfrsf12a were significantly upregulated following TAA or APAP treatment (Fig. 1H), and serum level of TWEAK was also elevated compared to PBS-group (Fig. S1E–F), suggesting a potential role for TWEAK/Tnfrsf12a axis during ALF. In summary, these findings demonstrate that oxidative stress, DNA damage, and inflammation are induced during ALF, and the TWEAK/Tnfrsf12a axis might play a role in the pathogenesis of ALF.

### Inhibition of TWEAK/Tnfrsf12a axis attenuates ALF induced by TAA or APAP

**Fig. 2:**
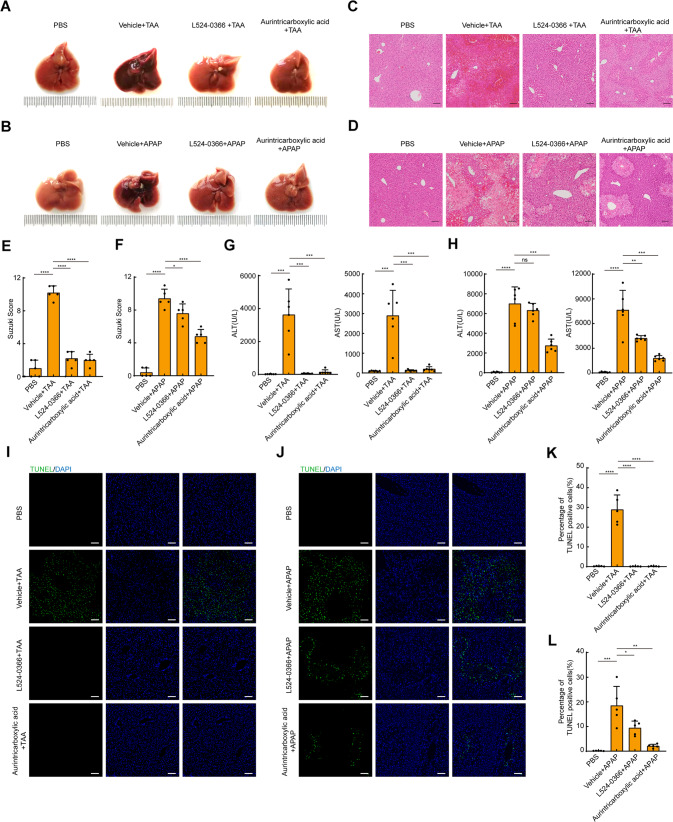
Inhibitors of TWEAK/Tnfrsf12a axis attenuates ALF induced by TAA or APAP. **A**, **B** Representative macroscopic appearance of livers at 24 h following TAA or APAP treatment in the presence or absence of TWEAK/Tnfrsf12a axis inhibitors. **C**, **D** Representative H&E-stained liver sections. Scale bars, 100 μm. **E**, **F** Suzuki score of H&E-stained liver sections (*n* = 5; mean ± s.d.). *P* values were determined by Student’s *t* test (*P < 0.05; ****P < 0.0001). **G**, **H** Serum levels of ALT and AST(U/L) (*n* = 6; mean ± s.d.). *P* values were determined by Student’s *t* test (**P < 0.01; ***P < 0.001; ****P < 0.0001; ns, not significant). **I**, **J** Representative TUNEL-stained liver sections. Scale bars, 100 μm. **K**, **L** Percentage of TUNEL positive cells in liver sections (*n* = 5; mean ± s.d.). *P* values were determined by Student’s *t* test (*P < 0.05; **P < 0.01; ***P < 0.001; ****P < 0.0001). All experiments were repeated at least two times.

To investigate whether TWEAK/Tnfrsf12a axis was involved in the pathogenesis of ALF, we used L524-0366 [9] and aurintricarboxylic acid [10] (two specific inhibitors of TWAEK/Tnfrsf12a axis) to explore the role of TWEAK/Tnfrsf12a axis during ALF. Mice were pretreated with the inhibitors or vehicle buffer prior to TAA or APAP administration. Sterilized PBS alone was used as the negative control. Besides, to avoid the protective effects of DMSO for APAP [11, 12], inhibitors were formulated in PEG400. Compared to the vehicle control group, pretreatment with L524-0366 or aurintricarboxylic acid significantly inhibited ALF (Fig. 2A, B). Histologically, the hepatic lobules exhibited centrilobular necrosis with hemorrhage after TAA or APAP administration. However, liver damage area was markedly reduced in the presence with L524-0366 or aurintricarboxylic acid (Fig. 2C–F). Moreover, mice pretreated with L524-0366 or aurintricarboxylic acid also exhibited lower serum ALT and AST than the vehicle control group (Fig. 2G, H). In addition, percentage of TUNEL staining cells in L524-0366- or aurintricarboxylic acid-pretreated mice were also decreased compared to the vehicle group, suggesting less DNA damage (Fig. 2I–L). Despite a significant protective effect of DMSO against APAP-induced liver injury, we still observed that pretreatment with L524-0366 or aurintricarboxylic acid dissolved in 2% DMSO significantly attenuated liver injury induced by APAP compared to the vehicle control group (Fig. S4A–D). In summary, these results demonstrate that TWEAK/Tnfrsf12a axis plays a crucial role in the pathogenesis of ALF, and inhibition of TWEAK/Tnfrsf12a axis has a protective effect against ALF.

### TWEAK/Tnfrsf12a axis induces RIPK1-dependent apoptosis of hepatocytes in a proinflammatory environment

**Fig. 3:**
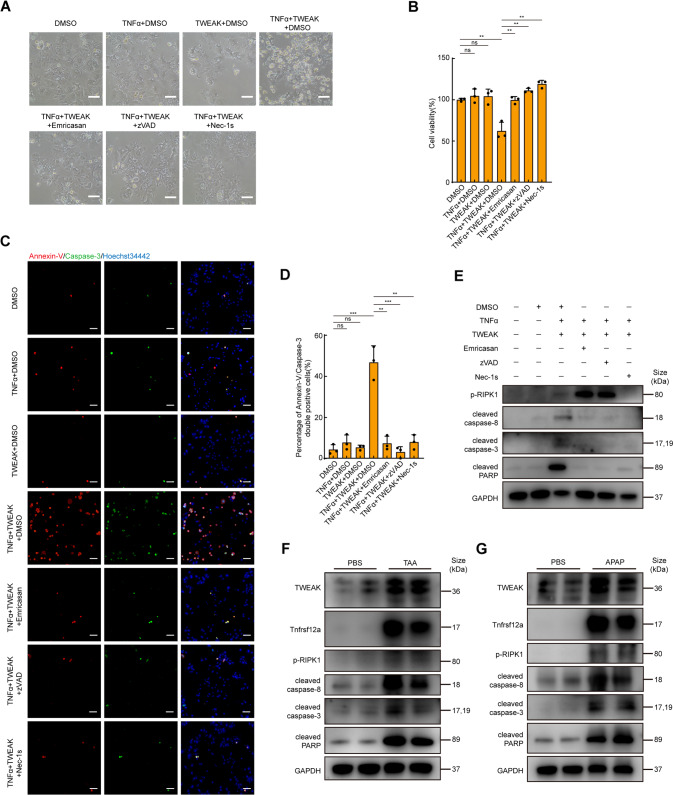
TWEAK/Tnfrsfs12a axis induces RIPK1-dependent apoptosis in a proinflammatory environment in hepatocytes. **A** Morphology of hepatocytes following TWEAK and TNFα stimulation at 24 h in the presence or absence of cell death inhibitors. Scale bar, 100 μm. **B** Quantification of cell viability of **A** (*n* = 3; mean ± s.d.). *P* values were determined by Student’s *t* test (**P < 0.01; ns, not significant). **C** Assessment of Annexin-V/Caspase-3 double positive cells stimulated following TWEAK and TNFα stimulation at 24 h. Scale bar, 100 μm. **D** Percentage of Annexin-V/Caspase-3 double positive cells of C (*n* = 3; mean ± s.d.). *P* values were determined by Student’s *t* test (**P < 0.01; ***P < 0.001; ns, not significant). **E** Expression of phospho-RIPK1 (p-RIPK1), cleaved caspase-8, cleaved caspase-3, and cleaved PARP of hepatocytes were analyzed with corresponding antibodies following TWEAK and TNFα stimulation at 12 h. GAPDH was used for normalization. **F**, **G** Immunoblotting analysis of whole liver lysates following TAA or APAP treatment at 24 h with corresponding antibodies, *n* = 2 per group. GAPDH was used for normalization. All experiments were repeated at least two times.

To explore the molecular mechanism by which TWEAK/Tnfrsf12a axis mediated ALF, we next treated isolated primary hepatocytes with TWEAK. However, treatment with TWEAK did not contribute to any morphological changes of hepatocytes (Fig. 3A). The inflammatory cytokine TWEAK has been reported to induce cell death through RIPK1 in a proinflammatory environment [13], but whether this cellular response exists in the liver remains unknown. Thus, we additionally treated hepatocytes with TNFα. Although treatment with TNFα alone had no effects on hepatocytes, TWEAK and TNFα synergistically induced cell death of hepatocytes with canonical apoptosis morphology (Fig. 3A). Nec-1s [14], zVAD [15] or emricasan [16] (effective inhibitors for RIPK1 and caspase, respectively) inhibited cell death induced by TWEAK/TNFα (Fig. 3A, B). Consistent with the previous results, activation of caspase-3 and Annexin-V were also observed by fluorescence staining (Fig. 3C, D). Notably, treatment with zVAD or emricasan did not switch apoptosis to necroptosis in hepatocytes. Phosphorylation of RIPK1 was used as a marker of activation. RIPK1 phosphorylation was induced following TWEAK/TNFα treatment and significantly upregulated in the presence of zVAD or emricasan, but was not observed with Nec-1s (Fig. 3E). Nevertheless, such highly phosphorylation of RIPK1 did not induce necroptosis in hepatocytes in the presence of pan-caspase inhibitors. In addition, Nec-1s, zVAD, and emricasan prevented caspase-8, caspase-3, and PARP processing, but neither zVAD nor emricasan inhibited RIPK1 phosphorylation, indicating that TWEAK/TNFα-induced RIPK1 activation upstream of caspase-8, caspase-3, and PARP processing (Fig. 3E). Thus, hepatocytes undergo RIPK1-dependent apoptosis following TWEAK/TNFα treatment. Notably, similar results were also observed in vivo (Fig. 3F, G). Besides, KEGG and GSEA analysis also suggested that apoptosis was induced in the pathogenesis of ALF (Fig. 1B–D). Taken together, these data suggest that TWEAK/Tnfrsf12a axis activates RIPK1 and induces RIPK1-dependent apoptosis of hepatocytes in a proinflammatory environment.

### Inhibition of RIPK1 kinase activity prevents ALF induced by TAA or APAP

**Fig. 4:**
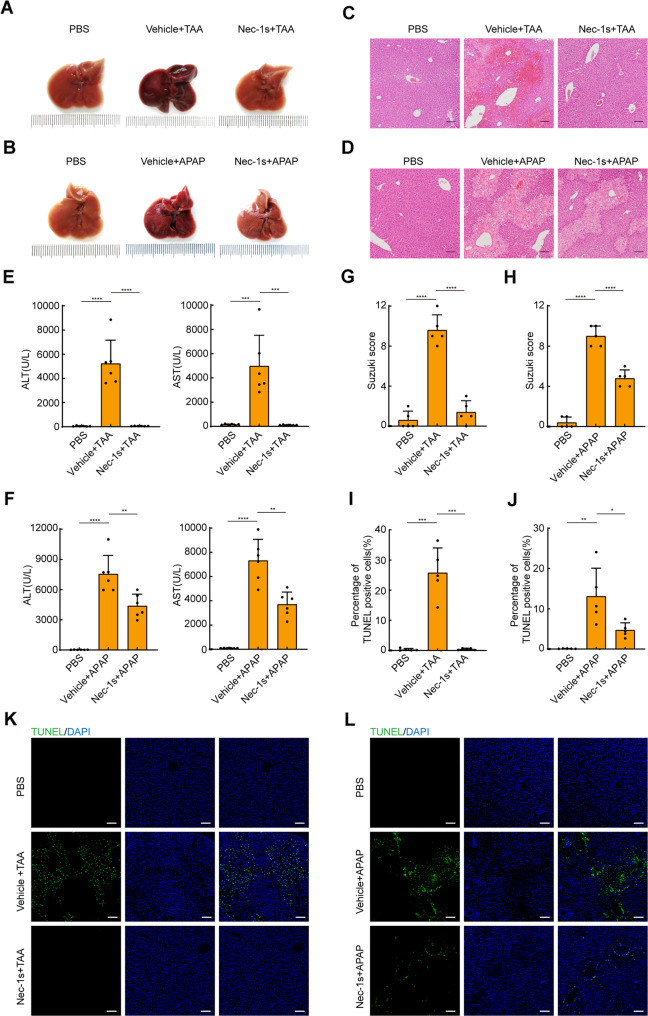
Nec-1s protects mice against ALF induced by TAA or APAP. **A**, **B** Representative macroscopic appearance of livers at 24 h following TAA or APAP treatment in the presence or absence of Nec-1s. **C**, **D** Representative H&E-stained liver sections. Scale bars, 100 μm. **E**, **F** Serum levels of ALT and AST(U/L) (*n* = 6; mean ± s.d.). *P* values were determined by Student’s *t* test (**P < 0.01; ***P < 0.001; ****P < 0.0001). **G**, **H** Suzuki score of H&E-stained liver sections (*n* = 5; mean ± s.d.). *P* values were determined by Student’s *t* test (****P < 0.0001). **I**, **J** Percentage of TUNEL positive cells in liver sections (*n* = 5; mean ± s.d.). *P* values were determined by Student’s *t* test (*P < 0.05; **P < 0.01; ***P < 0.001). **K**, **L** Representative TUNEL-stained liver sections. Scale bars, 100 μm. All experiments were repeated at least two times.

Activation of RIPK1 has been implicated during ALF [17–20], and our results suggested that TWEAK/Tnfrsf12a axis mediated ALF through RIPK1 activation. Therefore, we next investigated whether inhibition of RIPK1 could protect against ALF in these models. Mice were pretreated with Nec-1s for one hour and then induced ALF with TAA or APAP treatment. As expected, the macroscopic appearance of the liver showed dark red discoloration of the parenchyma in the vehicle control group, suggesting coagulation during ALF. However, pretreatment with Nec-1s rescued these pathological symptoms, suggesting an involvement of RIPK1 kinase activity in the pathogenesis of ALF (Fig. 4A, B). Histological changes in the liver were assessed by H&E. The hepatic lobules exhibited obvious centrilobular necrosis with hemorrhage after TAA or APAP treatment. In contrast, liver damage was significantly inhibited in the Nec-1s-pretreated group (Fig. 4C, D, G, H). In addition, serum levels of ALT and AST were decreased in the Nec-1s-pretreated group compared to the vehicle control group (Fig. 4E, F). DNA damage in the liver was detected by TUNEL staining. The percentage of TUNEL-positive cells in the Nec-1s-pretreated group was also lower than the vehicle control group (Fig. 4I–L). Similarly, Nec-1s dissolved in 2% DMSO also exhibited a protective effect against APAP compared to the vehicle control group (Fig. S4E–H). Altogether, these data provide powerful support for a critical role of RIPK1 in the pathogenesis of ALF.

### RIPK3 or MLKL deficiency does not protect against ALF induced by TAA or APAP

RIPK1 is a crucial upstream regulator of necroptosis, which is mediated by the RIPK1-RIPK3-MLKL signaling pathway [21]. Necroptosis has been implicated in many diseases ranging from ischemia/reperfusion injuries to Alzheimer’s disease [22]. Our results showed that pharmaceutical inhibition of RIPK1 exhibited great protective effects against ALF induced by TAA or APAP, raising the possibility that necroptosis was involved in the pathogenesis of ALF. Thus, to investigate whether RIPK1 regulated ALF through RIPK1-RIPK3-MLKL-dependent necroptosis, we treated RIPK3 knockout mice and their strain-matched controls with TAA or APAP, respectively (Fig. S7A). However, macroscopic appearance revealed that RIPK3 knockout mice exhibited severe liver injury (Fig. S2A–B). Moreover, histological staining (Fig. S2C–D and G–H) and serum levels of transaminases (Fig. S2E–F) in RIPK3 knockout mice were comparable to their strain-matched control mice, demonstrating that TAA or APAP-induced ALF does not depend on RIPK3. DNA damage was obvious as assessed by TUNEL staining in all groups (Fig. S2I–L). Therefore, our results demonstrate that RIPK3 does not contribute to ALF in these models.

Although we did not observe any protective effects of RIPK3 deficiency in these ALF models, RIPK1-MLKL-mediated necroptosis independently of RIPK3 had been implicated in other liver diseases [20]. Thus, to investigate whether RIPK1 regulated ALF through RIPK1-MLKL-mediated necroptosis, we treated MLKL knockout mice and strain-matched controls with TAA or APAP, respectively (Fig. S7B). However, MLKL deficiency also could not protect mice against TAA or APAP toxicity. Macroscopic liver damage across all groups were obvious (Fig. S3A–B). Histologically, the hepatic lobules showed extensive necrotic areas (Fig. S3C–D, G–H), and serum levels of ALT and AST were dramatically elevated in all groups (Fig. S3E–F). DNA damage was also widely distributed around the centrilobular area, as assessed by TUNEL staining (Fig. S3I–L). Taken together, our work demonstrates that RIPK1 regulates cell death in the pathogenesis of ALF independent of the RIPK1-RIPK3-MLKL signaling pathway.

### Inhibition of apoptosis through a pan-caspase inhibitor prevents ALF induced by TAA or APAP

**Fig. 5:**
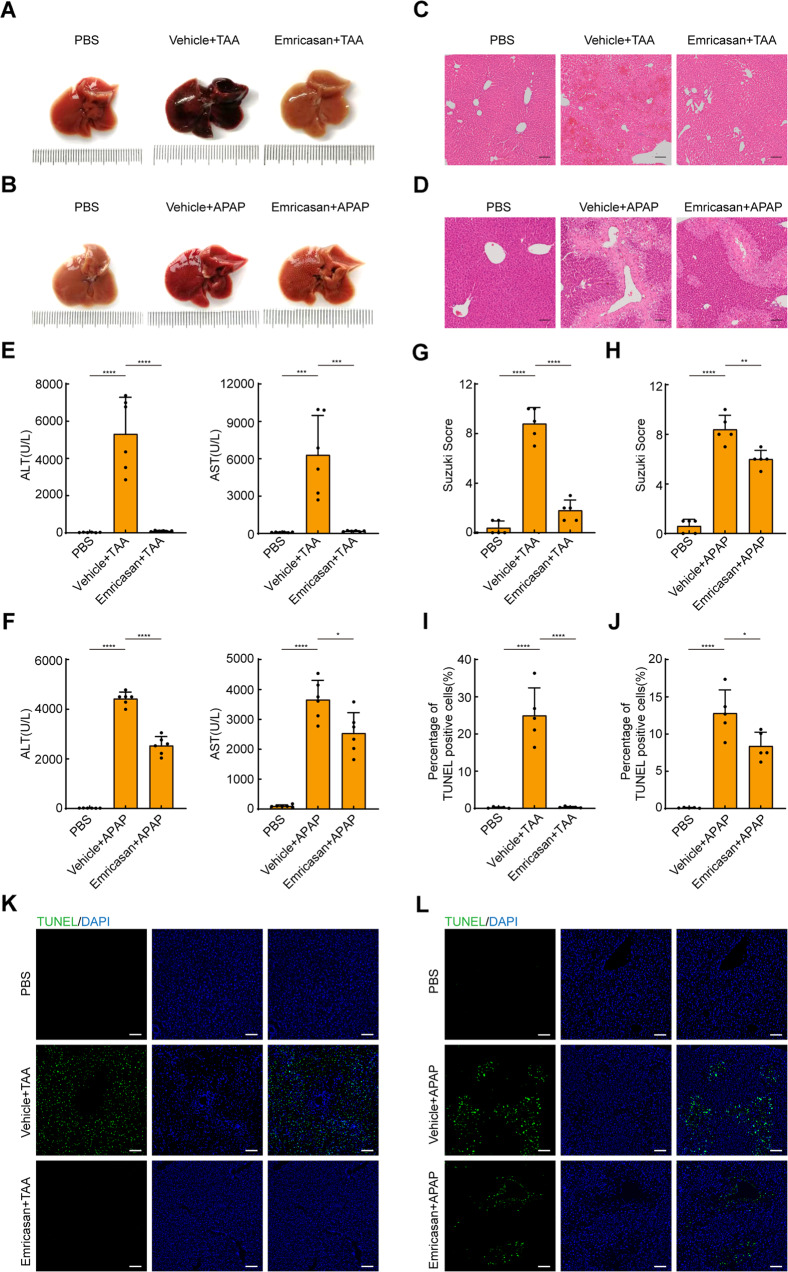
Emricasan attenuates ALF induced by TAA or APAP. **A**, **B** Representative macroscopic appearance of livers at 24 h following TAA or APAP treatment in the presence or absence of emricasan. **C**, **D** Representative H&E-stained liver sections. Scale bars, 100 μm. **E**, **F** Serum levels of ALT and AST(U/L) (*n* = 6; mean ± s.d.). *P* values were determined by Student’s *t* test (*P < 0.05; ***P< 0.001; ****P < 0.0001). **G**, **H** Suzuki score of H&E-stained liver sections (*n* = 5; mean ± s.d.). *P* values were determined by Student’s t test (**P < 0.01; ****P < 0.0001). **I**, **J** Percentage of TUNEL positive cells in liver sections (*n* = 5; mean ± s.d.). *P* values were determined by Student’s *t* test (*P < 0.05; ****P < 0.0001). **K**, **L** Representative TUNEL-stained liver sections. Scale bars, 100 μm. All experiments were repeated at least two times.

Our previous results suggested that RIPK1-dependent apoptosis, but not RIPK1-dependent necroptosis, mediated ALF. To investigate whether inhibition of apoptosis protected mice from ALF, emricasan was injected one hour prior to TAA or APAP administration. As hypothesized, pretreatment with emricasan significantly attenuated liver damage in these ALF models (Fig. 5A, B). Specifically, histological staining revealed that pretreatment with emircasan reduced necrotic areas compared to the vehicle group (Fig. 5C, D, G, H). In addition, serum levels of ALT and AST were significantly decreased in emricasan-pretreated group (Fig. 5E, F). DNA damage was also inhibited in the presence of emricasan (Fig. 5I–L). Emricasan that was dissolved in 2% DMSO also prevented liver injury induced by APAP in contrast to vehicle control group (Fig. S4I–L). Collectively, these results further confirm that RIPK1 and pan-caspase inhibitors significantly attenuate ALF induced by TAA or APAP, suggesting TWEAK/Tnfrsf12a axis induces RIPK1-dependent apoptosis and subsequent liver injury.

### Neither GSDMD nor GSDME knockout inhibit ALF induced by TAA or APAP

As described, both Nec-1s and emricasan can effectively inhibit ALF, demonstrating that RIPK1-dependent apoptosis contributed to ALF induced by TAA or APAP. However, canonical apoptosis is generally considered as non-inflammatory without leakage of cell content due to intact cell membrane [23]. Normally, apoptotic cells are phagocytosed in vivo by scavenger cells such as macrophages. However, if apoptotic cells are not removed timely at the terminal stage following the completion of an apoptotic program, they will progress to a phase called secondary necrosis, which is characterized by osmotic cell swelling and lysis [24, 25]. Considering that ALF is characterized by extensive necrosis, we hypothesized that another mode of lytic cell death might occur in these ALF models.

Recent studies have shown that gasdermins, especially GSDMD and GSDME, can be activated by caspase cleavage, leading to pyroptosis or secondary necrosis [26–30]. Therefore, activation of caspase in these murine ALF models might cleave GSDMD or GSDME to induce pyroptosis, causing necroinflammation and liver injury. To investigate whether ALF was mediated by GSDMD or GSDME, we treated GSDMD or GSDME knockout mice and strain-matched controls with TAA or APAP, respectively (Fig. S7C–D). Macroscopic appearance of livers showed severe liver injury in all groups of mice (Figs. S5A–B and S6A–B). Histologically, the hepatic lobules exhibited extensive centrilobular necrosis with hemorrhage in all groups (Figs. S5C–D and S6C–D). Suzuki score of liver sections showed similar results (Figs. S5G–H and S6G–H). There was also no difference in the levels of serum transaminases (Figs. S5E–F and S6E–F). Moreover, the percentage of TUNEL-positive cells dramatically increased across all groups of mice (Figs. S5I–L and S6I–L), suggesting extensive DNA damage. However, it is possible that both GSDMD and GSDME were involved in the pathogenesis of ALF, and ALF only could be alleviated by knockout of GSDMD and GSDME simultaneously. Cleavage of gasdermins was used as a marker of activation, but cleavage of GSDMD or GSDME were not observed during ALF (Fig. S7E). Although a small size band was observed using GSDME antibody, it decreased following TAA treatment and did not match the size of GSDME cleavage product, suggesting non-specific bands. In summary, these findings demonstrate that neither GSDMD nor GSDME deficiency protect mice from ALF, and provide us with a reasonable assumption that following TAA or APAP treatment, TWEAK/Tnfrsf12a axis activates RIPK1 and induces an excessive number of apoptotic cells that beyond the capabilities of scavenger cells, which in turn resulting in secondary necrosis and ultimate ALF.

### Induced expression of TNFRSF12A and TWEAK in patients with ALF

To investigate whether the noted involvement of TWEAK/TNFRSF12A axis in animal models of ALF could be observed in human patients, we analyzed samples from a publicly available human dataset of ALF patients from the GEO (GSE74000) [31]. Remarkably, expression of hepatic TWEAK and TNFRSF12A were significantly upregulated in ALF patients compared to normal liver donors (Fig. S8A–B). Thus, these results demonstrate that expression of hepatic TWEAK and TNFRSF12A are induced in ALF patients, and targeting the TWEAK/TNFRSF12A axis may be a promising therapeutic target for ALF in the clinic.

## Discussion

Acute liver failure (ALF) is characterized by rapid loss of liver function, accompanied by coagulopathy and hepatic encephalopathy. It is usually caused by acute viral infection or hepatotoxic substances, such as APAP overdose. Due to unknown molecular mechanisms and lack of therapeutic targets, the most effective treatment for ALF is currently limited to liver transplantation. Therefore, to identify novel therapies for ALF, numerous studies have been performed to explore the regulatory mechanism of ALF.

Of these, the abnormal death of liver cells is the leading cause that drives the severity and outcome of almost every hepatic disease. Accidental and unregulated necrosis has long been regarded as the ultimate consequence of extreme physical stress or chemical toxicity, but the concept of programmed cell death has changed this opinion in recent years. For example, several studies based on a variety of experimental approaches showed that RIPK1-dependent necroptosis was involved in the pathogenesis of ALF [19, 20, 32]. However, these could not be confirmed by genetic approaches in these same experimental models [18, 33, 34].

In this study, we show that TWEAK/Tnfrsf12a axis is significantly upregulated in murine ALF models. TWEAK/Tnfrsf12a axis has been shown to induce RIPK1-dependent necroptosis and acute kidney injury [7, 8], suggesting that ALF might be also mediated by TWEAK/Tnfrsf12a axis and RIPK1-dependent necroptosis. Indeed, we provide compelling evidence that inhibition of TWEAK/Tnfrsf12a axis significantly attenuates ALF, and a synergism of TWEAK and TNFα triggers RIPK1-dependent apoptosis of hepatocytes in vitro. However, pan-caspase inhibitors do not switch apoptosis to necroptosis in this case. Furthermore, both RIPK1 and pan-caspase inhibitors can inhibit ALF in vivo. This observation is in accordance with the results of previous studies that hepatocytes did not express RIPK3 under basal conditions and blocking apoptosis in hepatocytes did not switch to necroptosis [35]. The undetectable expression of RIPK3 in liver was also confirmed by immunoblotting (Fig. S6A). As we all know, cells that do not express RIPK3 do not undergo necroptosis [36]. Therefore, these results explain why RIPK1-dependent cell death in TAA or APAP-induced ALF could be blocked by Nec-1s or emricasan, but not by knockout of RIPK3 or MLKL.

On the other hand, recent studies have shown that activated caspases can cleave gasdermins to induce pyroptosis, causing necroinflammation and injury [26–30]. The gasdermins family comprises six members in human: GSDMA, GSDMB, GSDMC, GSDMD, GSDME, and PJVK. However, unlike human, rodents lack GSDMB, and expression of GSDMA, GSDMC, and PJVK are undetected in livers of mice [37]. Therefore, only GSDMD and GSDME are likely to contribute to liver injury in mice. However, neither GSDMD nor GSDME deficiency could protect mice against TAA or APAP toxicity, strongly suggesting that ALF in these murine models is not mediated by pyroptosis. Besides, KEGG and GSEA analysis in transcriptome sequencing also suggested that apoptosis was involved in the pathogenesis of ALF. Taken together, these results provide compelling evidence that RIPK1-dependent apoptosis, but not necroptosis or pyroptosis, contributes to ALF induced by TAA or APAP.

Remarkably, the expression levels of hepatic TWEAK and TNFRSF12A are upregulated in patients with ALF when compared to normal donors, suggesting a potential role of TWEAK/TNFRSF12A axis in human ALF patients. Thus, the TWEAK/TNFRSF12A pathway may be a promising therapeutic target for ALF for clinical application. In addition, these cell death inhibitors tested in this study also exhibit great protective effects and are already in clinical trials test for other diseases. Considering that ALF treatment is currently limited to liver transplantation, translation of these inhibitors to the clinic as a potential treatment of ALF condition merits further research.

## Materials and methods

### Animals and housing of mice

C57BL/6J male mice were purchased from Charles River Laboratories (Shanghai, China). GSDMD knockout and GSDME knockout mice were purchased from GemPharmatech (Nanjing, China). RIPK3 knockout mice were kindly provided by Dr. Hongguang Xia of Zhejiang University school of medicine, Zhejiang. MLKL knockout mice were kindly provided by Dr. Liming Sun of Center for Excellence in Molecular Cell Science, University of Chinese Academy of Sciences, Shanghai. Male mice aged between 8 and 9 weeks were used for experiments. They were housed in a specific pathogen-free (SPF) environment under controlled conditions (22 °C–24 °C, 12/12 h light/dark cycle). The mice are allowed to acclimate to housing for seven days and randomized into different groups before experiments. The investigator was blinded to the group allocation of the animals during the experiment. Animals were fed standard laboratory chow and water. All animals care and experimental procedures in this study were evaluated and approved by the Institutional Animal Care and Use Committee of ShanghaiTech University under the document number of 20200629001 and following the internationally recognized guidelines on animal welfare.

### Animal model of acute liver failure

For the TAA-induced ALF model, all male mice (8–9 weeks old) were injected intraperitoneally with 200 mg/kg of TAA (sigma, 172502). For the APAP-induced ALF model, all male mice (8–9 weeks old) were fasted overnight and injected intragastrically with 500 mg/kg of APAP (meilunbio, MB1698). Fasting was maintained for 6 h after APAP treatment. The same volume of sterilized PBS was injected into fed or fasted mice as a control group for TAA or APAP-induced ALF, respectively. The sample size of each experiment is shown in the legend. For inhibitors treatment, L524-0366 (TargetMol, T8570, 10 mg/kg), aurintricarboxylic acid (MedChemExpress, HY-122575, 10 mg/kg), Nec-1s (Selleck, S8641, 2.5 mg/kg), emricasan (Selleck, S7775, 10 mg/kg) were injected one hour prior to TAA or APAP administration. All inhibitors were dissolved in 2%DMSO + 30%PEG400 + 5%Tween-80 + 63%ddH_2_O and injected intraperitoneally into mice. Of these, emricasan was injected twice daily, whereas others were injected only once daily. To avoid the protective effects by DMSO for APAP toxicity, we also dissolved these inhibitors in 30%PEG400 + 5%Tween-80 + 65%ddH_2_O and analyzed the effects of inhibitors without DMSO in APAP-induced ALF model. The same volume of vehicle buffer was injected to mice prior to TAA or APAP administration as a vehicle control group. To avoid known circadian effects on liver, all experiments were performed between 13:00 and 15:00. Serum and liver tissues were harvested 24 hours after TAA or APAP administration for analysis.

### Histopathology and TUNEL staining

For histological analysis, liver tissues were fixed in 4% paraformaldehyde and embedded in paraffin, then the sectioned tissues (3 μm) were stained with hematoxylin and eosin (H&E) kit (Sangon, E607318) or terminal deoxynucleotidyl transferase (TdT)-mediated dUTP nick-end labeling (TUNEL) kit (Vazyme, A112). H&E pictures were captured under Olympus VS120 light microscopy (Olympus, Japan) and the degree of liver damage was evaluated according to Suzuki score. TUNEL pictures were captured under Zeiss Axio Imager Z2 microscope (Zeiss, Germany).

### Serum transaminases and cytokines analysis

Serum levels of alanine aminotransferase (ALT) and aspartate aminotransferase (AST) were measured using a multiparametric analyzer (AU5811, Beckman Coulter, USA) according to an automated procedure. The serum levels of cytokines were measured by ELISA kits according to the manufacturer’s instructions. TNFα ELISA kit (BBI life sciences, D721026), IL-1β ELISA kit (BBI life sciences, D721017), IL-6 ELISA kit (BBI life sciences, D721022) and TWEAK ELISA kit (Cohesion Biosciences, CEK1569) were used according to the manufacturer’s instructions.

### Western blots assays

Frozen liver tissues or cells were treated with protein extraction reagent supplemented with protease inhibitor cocktail (Bimake, B14002). Extracted protein samples were used for electrophoresis and then transferred onto polyvinylidene difluoride (PVDF) membrane. The membranes were blocked with 5%(w/v) skim milk (BD Bioscience, USA) for 1 h at room temperature, and then incubated with the following primary antibodies overnight at 4 °C: TWEAK (Abcam, ab37170, 1:1000), Tnfrsf12a(Abcam, ab109365, 1:1000), Phospho-RIPK1 (Cell Signaling Technology, 31122S, 1:1000), cleaved caspase-8 (Cell Signaling Technology, 8592T, 1:1000), cleaved caspase-3 (Cell Signaling Technology, 9664S, 1:1000), cleaved PARP (Cell Signaling Technology, 94885S,1:1000), RIPK3 (Cell Signaling Technology, 15828, 1:1000), MLKL (Abgent, AP14272B, 1:1000), GSDMD (Abcam, ab219800, 1:1000), GSDME (Abcam, ab215191, 1:1000), Tubulin (Sigma, T6199, 1:1000), GAPDH (Proteintech, 10494-1-AP, 1:5000). Original western blots are presented in Supplementary file.

### Hepatocyte isolation and culture

Primary mouse hepatocytes were isolated by the standard two-step collagenase perfusion method [38]. Hepatocytes were purified by 40% Percoll (Sigma, P1644) and centrifugated at 1000 rpm/min for 10 min. The hepatocytes were suspended and seeded on culture plates with DMEM (Gibco, C11995500CP) supplemented with 10% of FBS (Gibco, 10091148), 1% Pen Strep (Solarbio, P1400). After hepatocytes were attached to the bottom of plates, the culture medium was changed to William’s E medium (Gibco, 12551032) containing 1% Glutamax (Gibco, 35050061), 1% Pen Strep, 1% insulin-transferrin-selenium (BasalMedia, S450) and 0.1 μM dexamethasone (Sigma, D4902). Primary hepatocytes were used for experiments within 2–3 days after isolation.

### Cell death assessment

After cells were attached, hepatocytes were treated with cytokines to induce cell death. The concentrations of the cytokine mixture were 100 ng/mL TWEAK (Sino Biological, 50174-M15H) and 50 ng/mL TNFα. For cell death inhibitors in vitro, 10 μM zVAD (Selleck, S7023), 10 μM Nec-1s (Selleck, S8641), 10 μM emricasan (Selleck, S7775) were dissolved in DMSO and added simultaneously with cytokines stimulation. After 24 hours, cell viability was measured by CellTiter-Lumi™ Plus (Beyotime, C0068L) according to the manufacturer’s instructions. To assess apoptotic cells, the cell was stained by Annexin-V/Caspase-3 kit (Beyotime, C1077M) according to the manufacturer’s instructions.

### qPCR and transcriptome sequencing

RNA was extracted using Trizol reagent (Thermo, 15596018). For qPCR experiment, cDNA was synthesized using HiScript III RT supermix (Vazyme, R323). qPCR was performed using ChamQ universal SYBR qPCR master mix (Vazyme, Q711). The primers used for qPCR were exhibited in Supplementary Table S1, actin was used for normalization. For transcriptome sequencing, 1 μg of total RNA was used for the construction of sequencing libraries and the transcriptome sequencing was performed using the high throughput sequencing platform of Illumina.

### Statistical analysis of a human dataset of ALF patients

Data of ALF patients were from a publicly available human dataset of the Gene Expression Omnibus (GEO; accession number GSE74000). GSE74000 consisted of three liver biopsies from patients suffering from APAP-induced ALF and two liver biopsies obtained from normal donors. Although this dataset contains few samples, it represents differential gene expression in human at the acute phase of this disease.

### Statistical analysis

Statistics and graphs were generated by GraphPad Prism 8. Results were shown as mean ± s.d. The variance within each group is similar. Group comparisons were performed using Student’s *t* test. *P* < 0.05 was considered statistically significant; ns, not significant; *P < 0.05; **P < 0.01; ***P < 0.001; ****P < 0.0001.

## Supplementary information

Supplementary Figures

Supplementary Table S1

Original Data File

Agreement with the change of authorship

## Data Availability

The raw data files for the sequencing analysis that generated in this study are deposited in the NCBI Gene Expression Omnibus (GEO) under the accession number GSE206595 (RNA-seq).

## References

[1] Lee WM, Squires RH, Nyberg SL, Doo E, Hoofnagle JH (2008). Acute liver failure: Summary of a workshop. Hepatology..

[2] Lee WM (2004). Acetaminophen and the U.S. Acute Liver Failure Study Group: lowering the risks of hepatic failure. Hepatology..

[3] Wiley SR, Winkles JA (2003). TWEAK, a member of the TNF superfamily, is a multifunctional cytokine that binds the TweakR/Fn14 receptor. Cytokine Growth Factor Rev.

[4] Locksley RM, Killeen N, Lenardo MJ (2001). The TNF and TNF receptor superfamilies integrating mammalian biology. Cell..

[5] Desplat-Jégo S, Varriale S, Creidy R, Terra R, Bernard D, Khrestchatisky M (2002). TWEAK is expressed by glial cells, induces astrocyte proliferation and increases EAE severity. J Neuroimmunol.

[6] Winkles JA (2008). The TWEAK-Fn14 cytokine-receptor axis: discovery, biology and therapeutic targeting. Nat Rev Drug Discov.

[7] Weinberg JM (2011). TWEAK-Fn14 as a mediator of acute kidney injury. Kidney Int.

[8] Martin-Sanchez D, Fontecha-Barriuso M, Carrasco S, Sanchez-Nino MD, Massenhausen AV, Linkermann A (2018). TWEAK and RIPK1 mediate a second wave of cell death during AKI. Proc Natl Acad Sci USA.

[9] Unudurthi SD, Nassal DM, Patel NJ, Thomas E, Yu J, Pierson CG (2020). Fibroblast growth factor-inducible 14 mediates macrophage infiltration in heart to promote pressure overload-induced cardiac dysfunction. Life Sci.

[10] Roos A, Dhruv HD, Mathews IT, Inge LJ, Tuncali S, Hartman LK (2017). Identification of aurintricarboxylic acid as a selective inhibitor of the TWEAK-Fn14 signaling pathway in glioblastoma cells. Oncotarget..

[11] Jeffery EH, Haschek WM (1988). Protection by dimethylsulfoxide against acetaminophen-induced hepatic, but not respiratory toxicity in the mouse. Toxicol Appl Pharm.

[12] Yoon MY, Kim SJ, Lee BH, Chung JH, Kim YC (2006). Effects of dimethylsulfoxide on metabolism and toxicity of acetaminophen in mice. Biol Pharm Bull.

[13] Vince JE, Chau D, Callus B, Wong WW, Hawkins CJ, Schneider P (2008). TWEAK-FN14 signaling induces lysosomal degradation of a cIAP1-TRAF2 complex to sensitize tumor cells to TNFalpha. J Cell Biol.

[14] Takahashi N, Duprez L, Grootjans S, Cauwels A, Nerinckx W, DuHadaway JB (2012). Necrostatin-1 analogues: critical issues on the specificity, activity and in vivo use in experimental disease models. Cell Death Dis.

[15] Slee EA, Zhu H, Chow SC, MacFARLANE M, Nicholson DW, COHEN GM (1996). Benzyloxycarbonyl-Val-Ala-Asp (OMe) fluoromethylketone (Z-VAD.FMK) inhibits apoptosis by blocking the processing of CPP32. Biochem J.

[16] Barreyro FJ, Holod S, Finocchietto PV, Camino AM, Aquino JB, Avagnina A (2015). The pan-caspase inhibitor Emricasan (IDN-6556) decreases liver injury and fibrosis in a murine model of non-alcoholic steatohepatitis. Liver Int.

[17] Yi Y, Zhang W, Tao L, Shao Q, Xu Q, Chen Y (2021). RIP1 kinase inactivation protects against acetaminophen-induced acute liver injury in mice. Free Radic Biol Med.

[18] Dara L, Johnson H, Suda J, Win S, Gaarde W, Han D (2015). Receptor interacting protein kinase 1 mediates murine acetaminophen toxicity independent of the necrosome and not through necroptosis. Hepatology..

[19] Takemoto K, Hatano E, Iwaisako K, Takeiri M, Noma N, Ohmae S (2014). Necrostatin-1 protects against reactive oxygen species (ROS)-induced hepatotoxicity in acetaminophen-induced acute liver failure. FEBS Open Bio.

[20] Gunther C, He GW, Kremer AE, Murphy JM, Petrie EJ, Amann K (2016). The pseudokinase MLKL mediates programmed hepatocellular necrosis independently of RIPK3 during hepatitis. J Clin Invest.

[21] Pasparakis M, Vandenabeele P (2015). Necroptosis and its role in inflammation. Nature..

[22] Mifflin L, Ofengeim D, Yuan J (2020). Receptor-interacting protein kinase 1 (RIPK1) as a therapeutic target. Nat Rev Drug Discov.

[23] Cohen GM (1997). Caspases: the executioners of apoptosis. Biochem J..

[24] Silva MT (2010). Secondary necrosis: the natural outcome of the complete apoptotic program. FEBS Lett.

[25] Silva MT, do Vale A, dos Santos NM (2008). Secondary necrosis in multicellular animals: an outcome of apoptosis with pathogenic implications. Apoptosis..

[26] Shi J, Zhao Y, Wang K, Shi X, Wang Y, Huang H (2015). Cleavage of GSDMD by inflammatory caspases determines pyroptotic cell death. Nature..

[27] Wang Y, Gao W, Shi X, Ding J, Liu W, He H (2017). Chemotherapy drugs induce pyroptosis through caspase-3 cleavage of a gasdermin. Nature..

[28] Rogers C, Fernandes-Alnemri T, Mayes L, Alnemri D, Cingolani G, Alnemri ES (2017). Cleavage of DFNA5 by caspase-3 during apoptosis mediates progression to secondary necrotic/pyroptotic cell death. Nat Commun.

[29] Orning P, Weng D, Starheim K, Ratner D, Best Z, Lee B (2018). Pathogen blockade of TAK1 triggers caspase-8-dependent cleavage of gasdermin D and cell death. Science..

[30] Sarhan J, Liu BC, Muendlein HI, Li P, Nilson R, Tang AY (2018). Caspase-8 induces cleavage of gasdermin D to elicit pyroptosis during Yersinia infection. Proc Natl Acad Sci USA.

[31] Rodrigues RM, Heymans A, De BV, Sachinidis A, Chaudhari U, Govaere O (2016). Toxicogenomics-based prediction of acetaminophen-induced liver injury using human hepatic cell systems. Toxicol Lett.

[32] Ramachandran A, McGill MR, Xie Y, Ni HM, Ding WX, Jaeschke H (2013). Receptor interacting protein kinase 3 is a critical early mediator of acetaminophen-induced hepatocyte necrosis in mice. Hepatology..

[33] Schneider AT, Gautheron J, Tacke F, Vucur M, Luedde T (2016). Receptor interacting protein kinase 1 (RIPK1) in hepatocytes does not mediate murine acetaminophen toxicity. Hepatology..

[34] Deutsch M, Graffeo CS, Rokosh R, Pansari M, Ochi A, Levie EM (2015). Divergent effects of RIP1 or RIP3 blockade in murine models of acute liver injury. Cell Death Dis.

[35] Dara L, Liu ZX, Kaplowitz N (2016). Questions and controversies: the role of necroptosis in liver disease. Cell Death Discov.

[36] Degterev A, Zhou W, Maki JL, Yuan J (2014). Assays for necroptosis and activity of RIP kinases. Methods Enzymol.

[37] Broz P, Pelegrin P, Shao F (2020). The gasdermins, a protein family executing cell death and inflammation. Nat Rev Immunol.

[38] Charni-Natan M, Goldstein I (2020). Protocol for primary mouse hepatocyte isolation. STAR Protoc.

