# Supplementary material for: Inhibition of TWEAK/Tnfrsf12a axis protects against acute liver failure by suppressing RIPK1-dependent apoptosis
Source: Cell Death Discov. 2022 Jul 19;8:328. doi: 10.1038/s41420-022-01123-0 (PMC9296540; doi:10.1038/s41420-022-01123-0)
Supplement: Supplementary file 1 — Supplementary Figures [file 41420_2022_1123_MOESM1_ESM.docx]

**
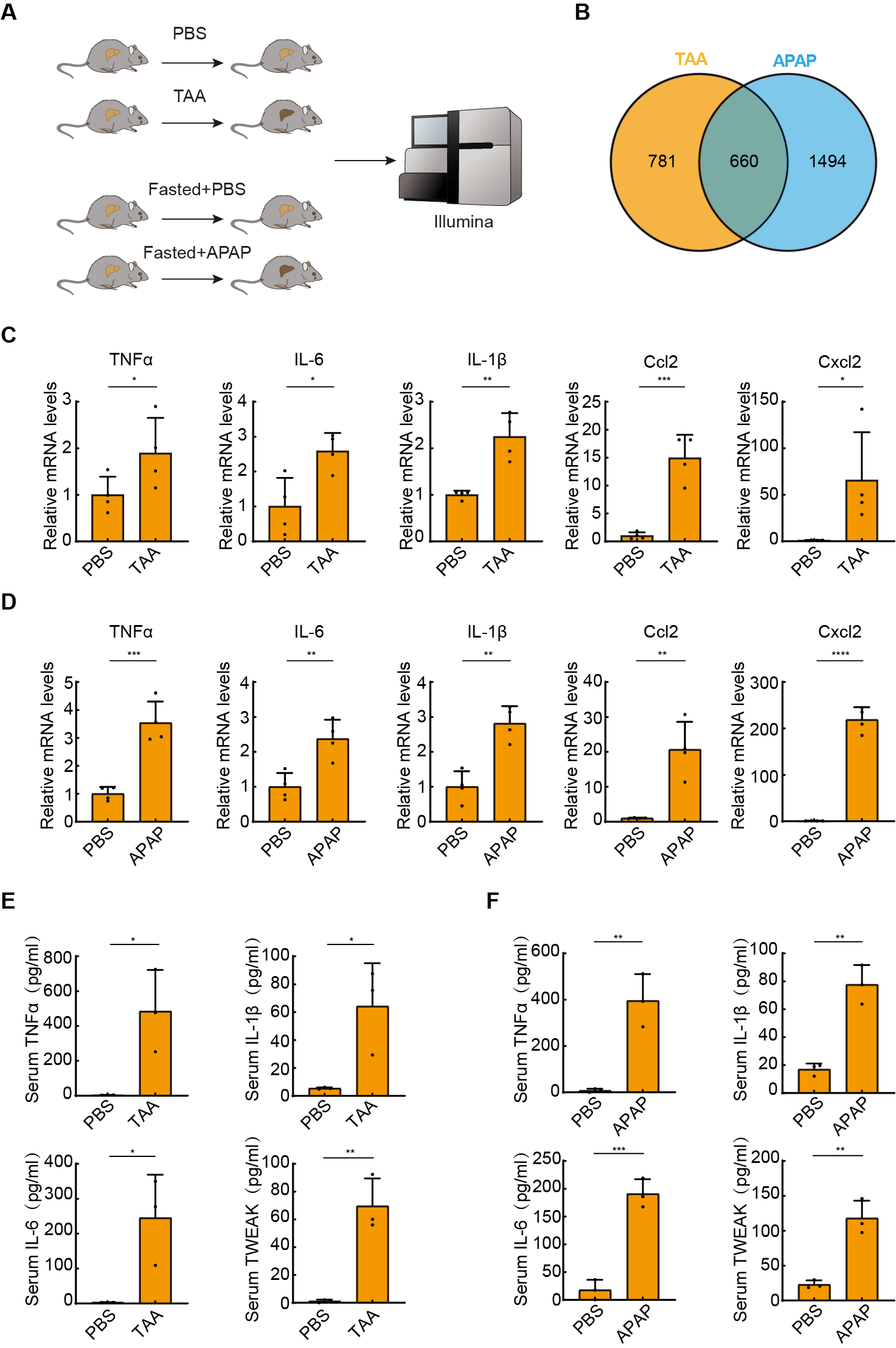
**

**Supplementary Figure S1: Inflammatory response is induced during ALF.**

(A) Experiment schematic. (B) Venn diagram showing overlap between sets of upregulated genes in ALF mice induced by TAA or APAP. (C&D) The mRNA expression levels of genes related to inflammation in livers of PBS-treated mice and ALF mice (n=4; mean ± s.d.). *P* values were determined by Student's t test (*, *P* < 0.05; **, *P* < 0.01; ***, *P* < 0.001; ****, *P* < 0.0001). (E&F) ELISA analysis of TNFα, IL-6, IL-1β and TWEAK (n=3; mean ± s.d.). *P* values were determined by Student's t test (*, *P* < 0.05; **, *P* < 0.01; ***, *P* < 0.001).

**
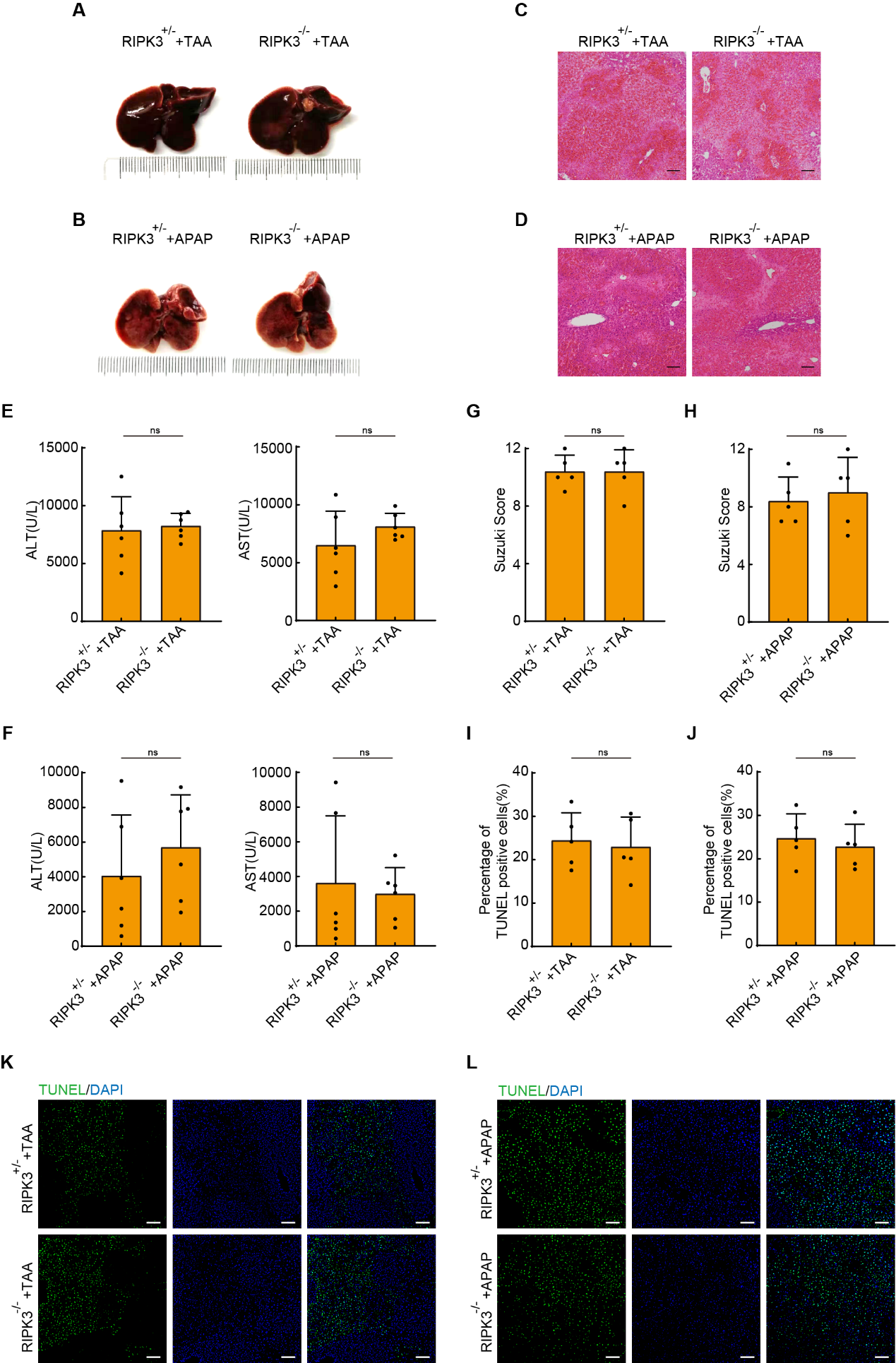
**

**Supplementary Figure S2:** **RIPK3 does not contribute to ALF induced by TAA or APAP.**

(A&B) Representative macroscopic appearance of livers of RIPK3 knockout mice and their strain-matched controls after TAA or APAP treatment. (C&D) Representative H&E-stained liver sections. Scale bars, 100 μm. (E&F) Serum levels of ALT and AST(U/L) (n=6; mean ± s.d.; ns, not significant). (G&H) Suzuki score of H&E-stained liver sections (n=5; mean ± s.d.; ns, not significant). (I&J) Percentage of TUNEL positive cells in liver sections (n=5; mean ± s.d.; ns, not significant). (K&L) Representative TUNEL-stained liver sections. Scale bars, 100 μm. All experiments were repeated at least two times.


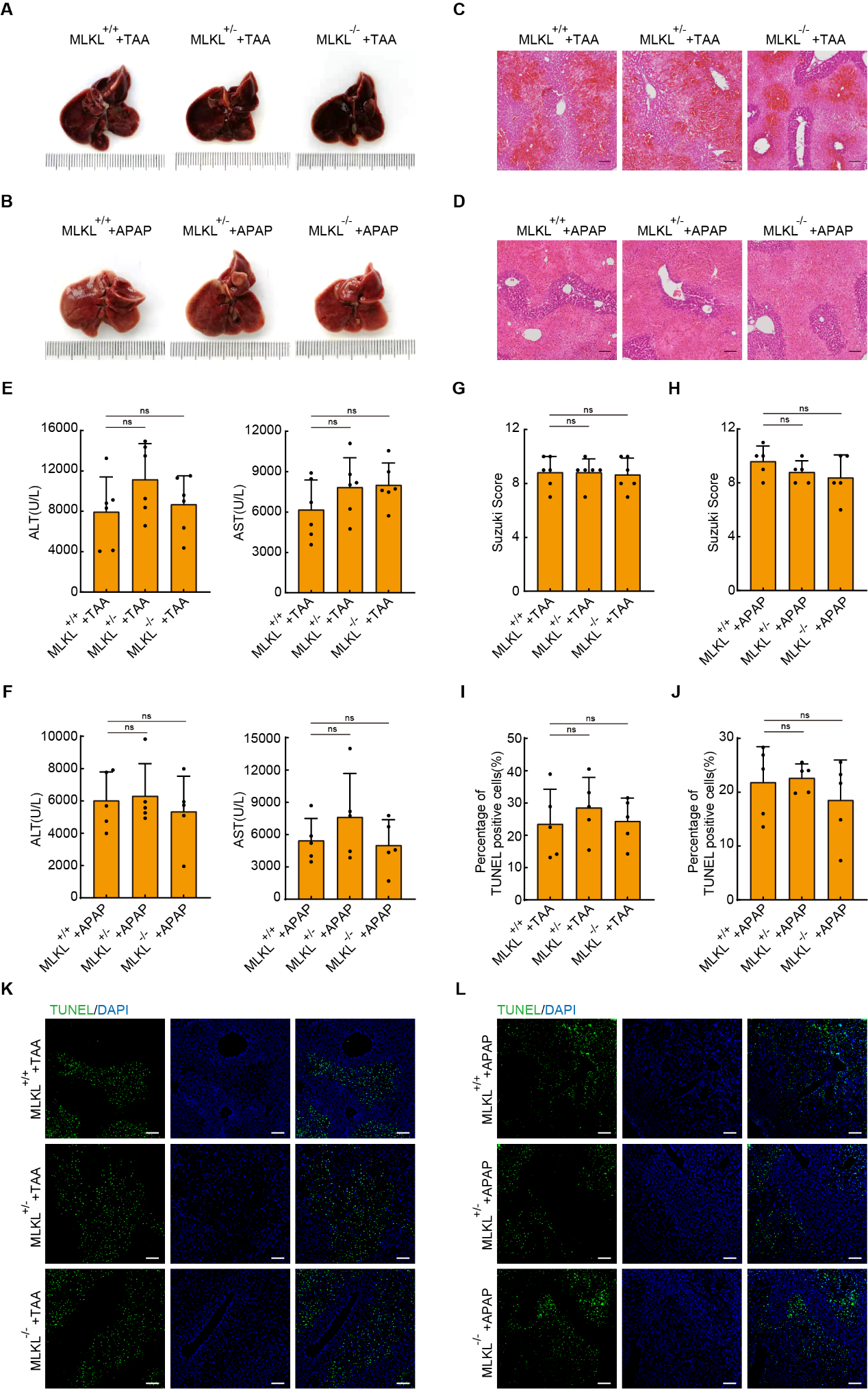


**Supplementary Figure S3: MLKL knockout does not inhibit ALF induced by TAA or APAP.**

(A&B) Representative macroscopic appearance of livers of MLKL knockout mice and their strain-matched controls after TAA or APAP treatment. (C&D) Representative H&E-stained liver sections. Scale bars, 100 μm. (E&F) Serum levels of ALT and AST(U/L) (n=6; mean ± s.d.; ns, not significant). (G&H) Suzuki score of H&E-stained liver sections (n=5; mean ± s.d.; ns, not significant). (I&J) Percentage of TUNEL positive cells in liver sections (n=5; mean ± s.d.; ns, not significant). (K&L) Representative TUNEL staining in liver sections. Scale bars, 100 μm. All experiments were repeated at least two times.

**
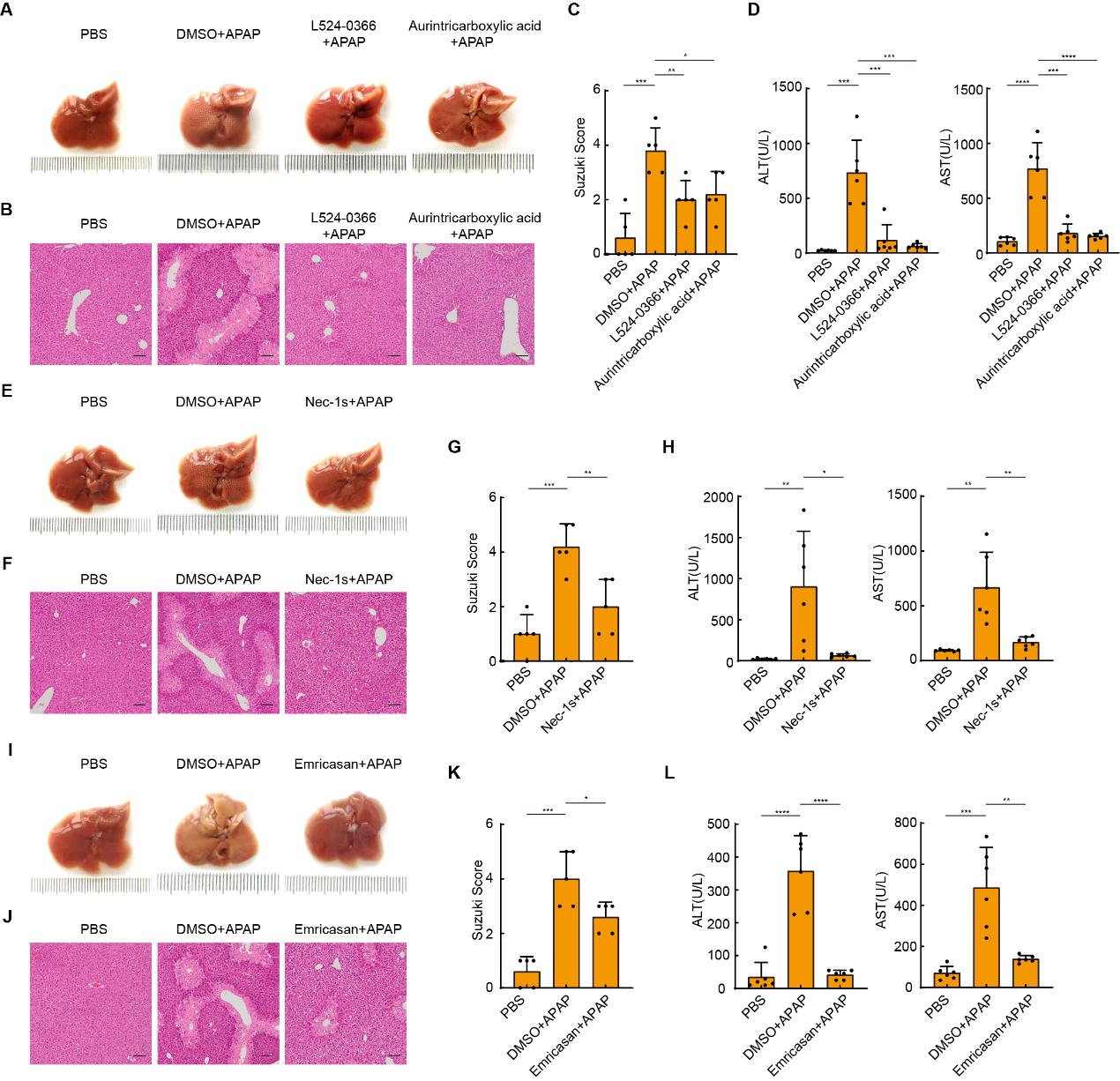
**

**Supplementary Figure S4: Inhibitors dissolved in DMSO exhibit protective effects against APAP-induced ALF.**

Indicated inhibitors were dissolved in 2%DMSO. Mice was pretreated with inhibitors or vehicle buffer one hour prior to APAP administration. (A, E, I) Representative macroscopic appearance of livers at 24 hours after APAP treatment with and without inhibitors. (B, F, J) Representative H&E-stained liver sections. Scale bars, 100 μm. (C, G, K) Suzuki score of H&E-stained liver sections (n=5; mean ± s.d.). *P* values were determined by Student's t test (*, *P* < 0.05; **, *P* < 0.01; ***, *P* < 0.001). (D, H, L) Serum levels of ALT and AST(U/L) (n=6; mean ± s.d.). *P* values were determined by Student's t test (*, *P* < 0.05; **, *P* < 0.01; ***, *P* < 0.001; ****, *P* < 0.0001). All experiments were repeated at least two times.


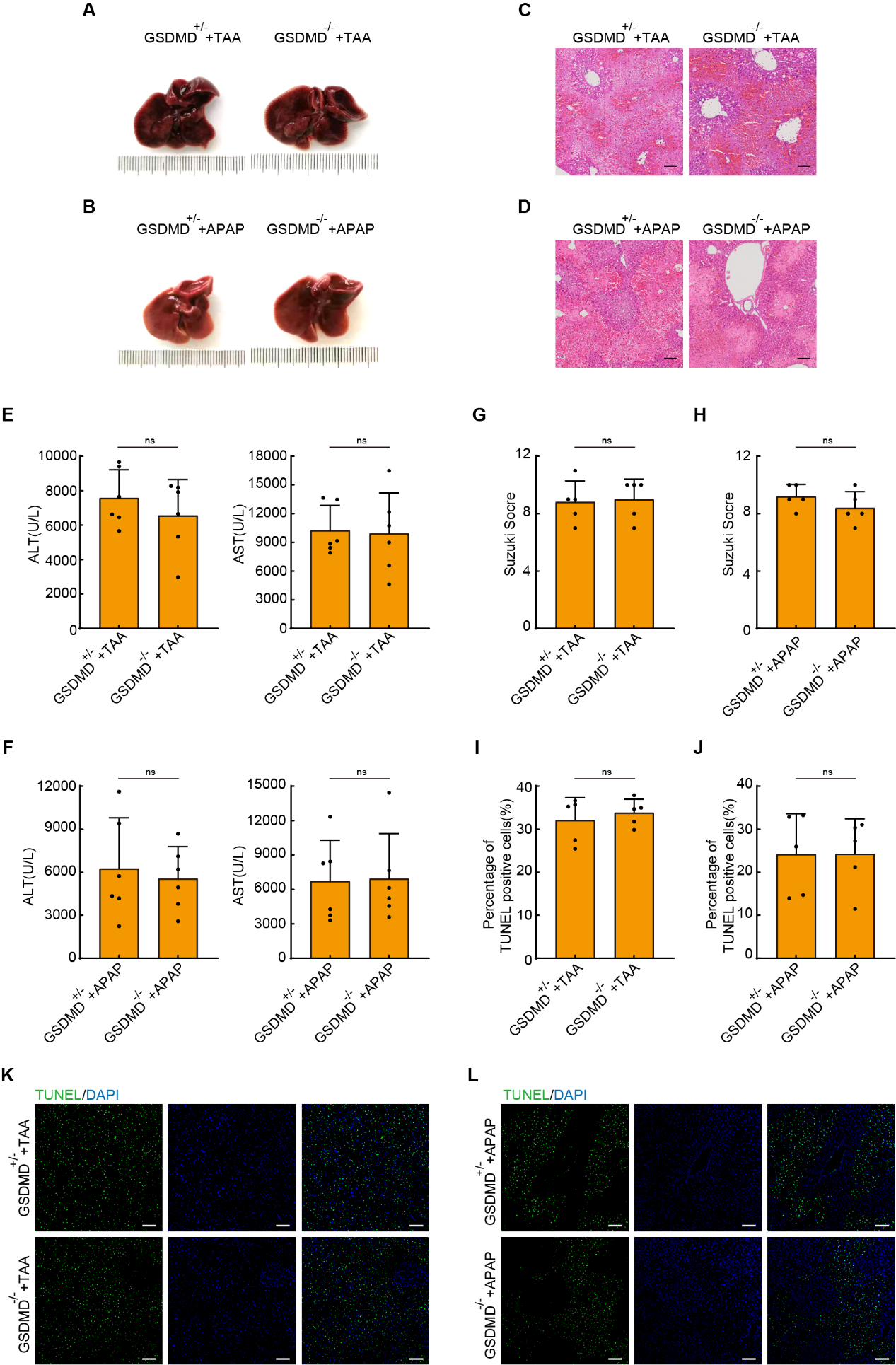


**Supplementary Figure S5: Knockout of GSDMD does not inhibit ALF induced by TAA or APAP**

(A&B) Representative macroscopic appearance of livers of GSDMD knockout mice and their strain-matched controls after TAA or APAP treatment. (C&D) Representative H&E-stained liver sections. Scale bars, 100 μm. (E&F) Serum levels of ALT and AST(U/L) (n=6; mean ± s.d.; ns, not significant) (G&H) Suzuki score of H&E-stained liver sections (n=5; mean ± s.d.; ns, not significant) (I&J) Percentage of TUNEL positive cells in liver sections (n=5; mean ± s.d.; ns, not significant) (K&L) Representative TUNEL-stained liver sections. Scale bars, 100 μm. All experiments were repeated at least two times.


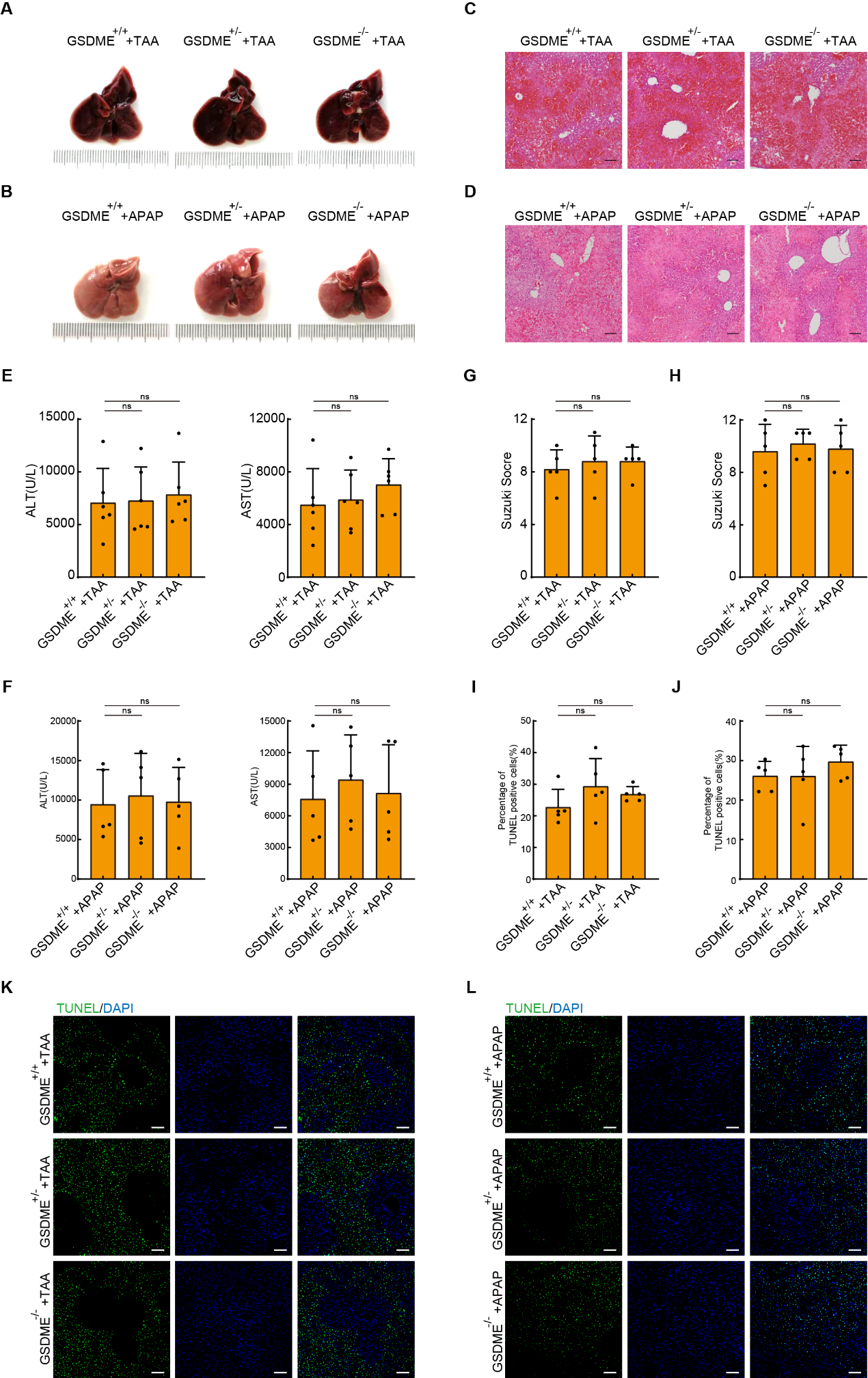


**Supplementary Figure S6: GSDME does not contribute to ALF induced by TAA or APAP**

(A&B) Representative macroscopic appearance of livers of GSDME knockout mice and their strain-matched controls after TAA or APAP treatment. (C&D) Representative H&E-stained liver sections of GSDME knockout mice and their strain-matched controls. Scale bars, 100 μm. (E&F) Serum levels of ALT and AST(U/L) (n=6; mean ± s.d.; ns, not significant) (G&H) Suzuki score of H&E-stained liver sections (n=5; mean ± s.d.; ns, not significant) (I&J) Percentage of TUNEL positive cells in liver sections (n=5; mean ± s.d.; ns, not significant) (K&L) Representative TUNEL staining in liver sections. Scale bars, 100 μm. All experiments were repeated at least two times.

**
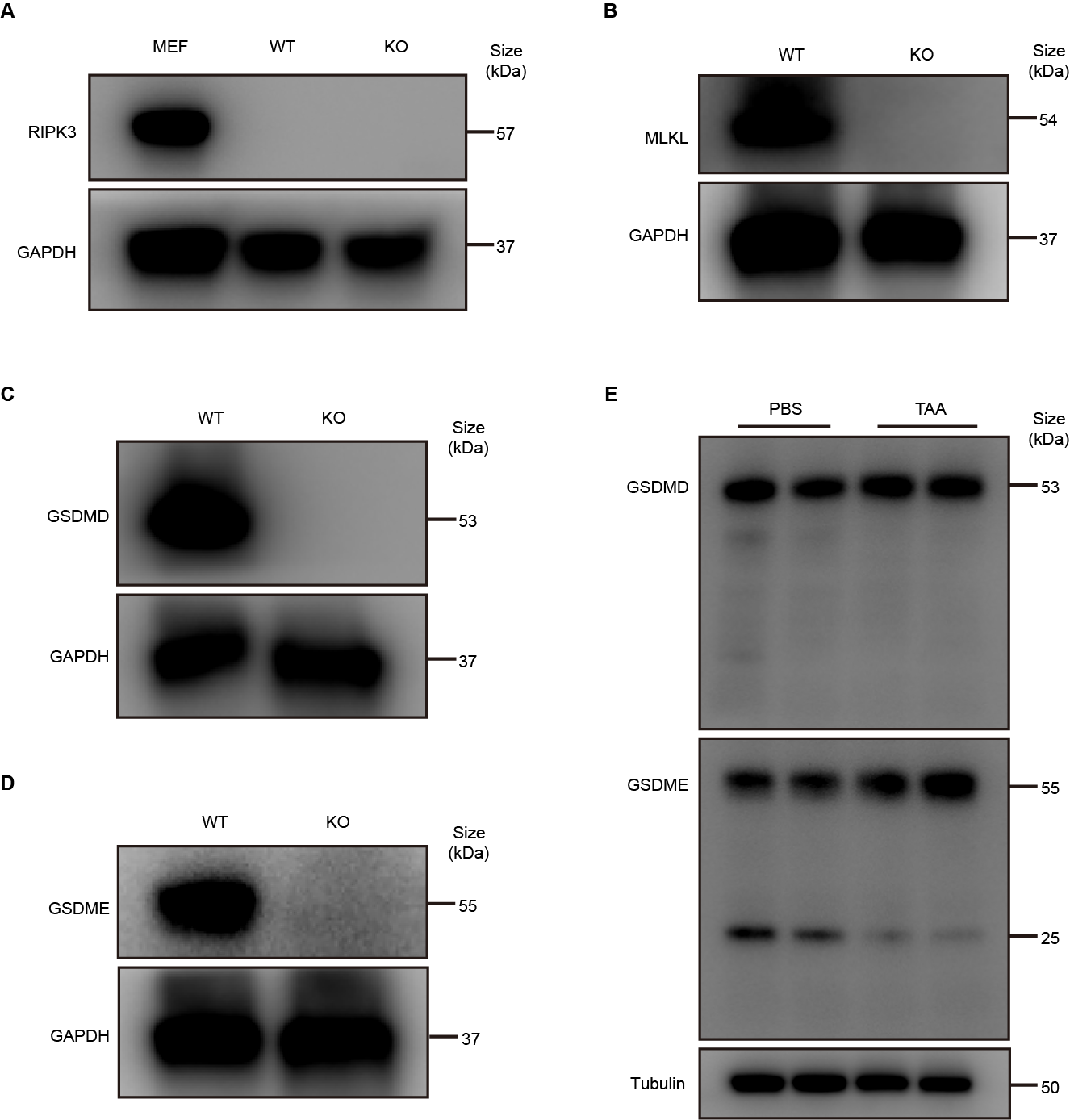
**

**Supplementary Figure S7: Western blot analysis of whole liver lysates with indicating antibodies.**

Verification of knockout mice by immunoblotting of liver lysates from C57 wide type (WT) mice and knockout (KO) mice with indicating antibodies. (A) RIPK3, mouse embryo fibroblast (MEF) was used as a positive control; (B) MLKL; (C) GSDMD; (D) GSDME. GAPDH was used for normalization. (E) Western blot analysis of whole liver lysates at 24 hours after TAA administration with corresponding antibodies, n=2 per group. Tubulin was used for normalization.


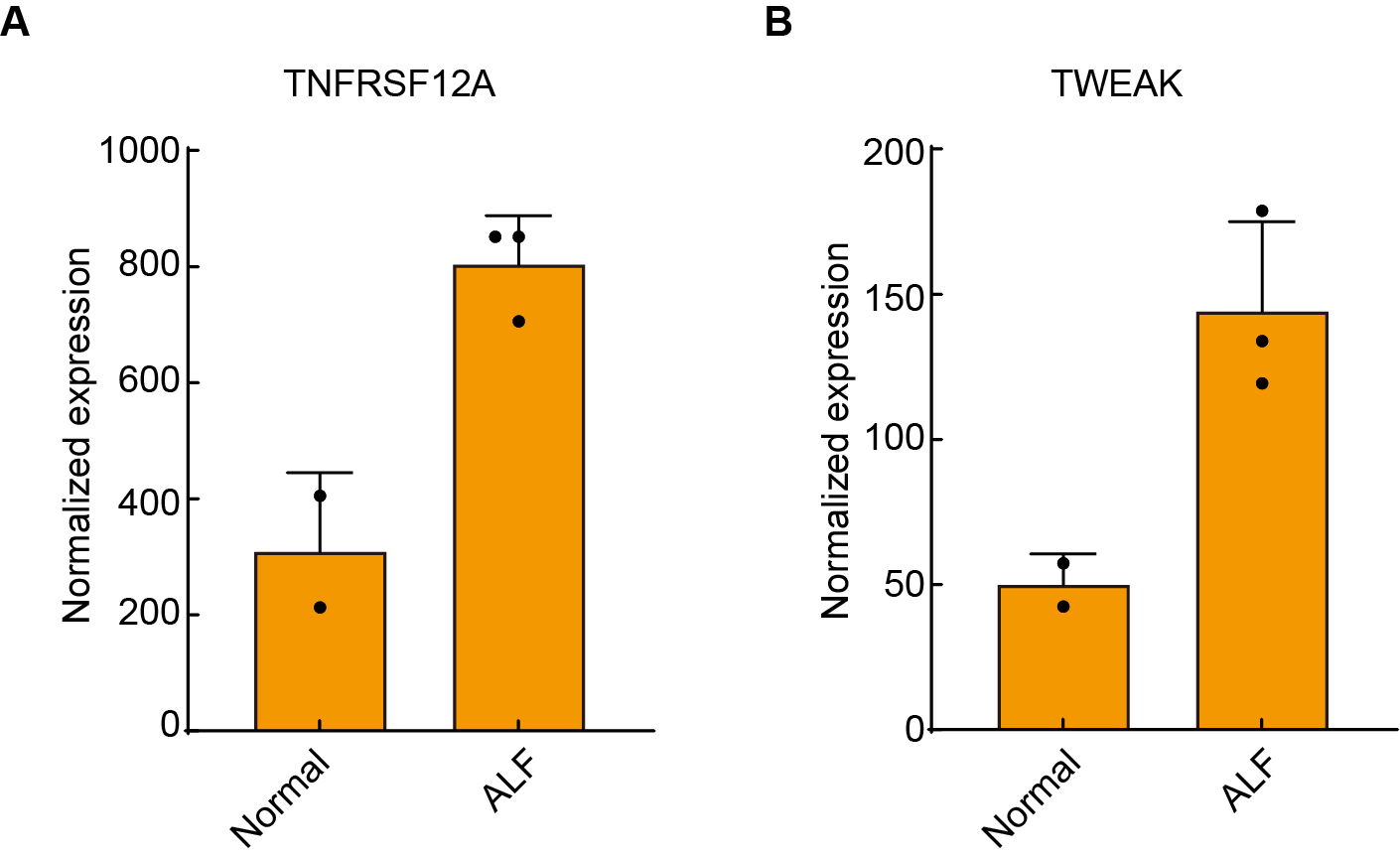


**Supplementary Figure S8: Increased expression of TNFRSF12A and TWEAK in patients with ALF**

Gene expression of TNFRSF12A (A) and TWEAK (B) in liver biopsies from patients suffering from APAP-induced ALF (n=3) and normal donor livers (n=2).

**Supplementary Table Legend (provided as excel form)**

**Supplementary Table S1**

The sequence information of qPCR primers for expression level detection.
