# Supplementary material for: Inhibition of TWEAK/Tnfrsf12a axis protects against acute liver failure by suppressing RIPK1-dependent apoptosis
Source: Cell Death Discov. 2022 Jul 19;8:328. doi: 10.1038/s41420-022-01123-0 (PMC9296540; doi:10.1038/s41420-022-01123-0)

Original image: Figure 1H

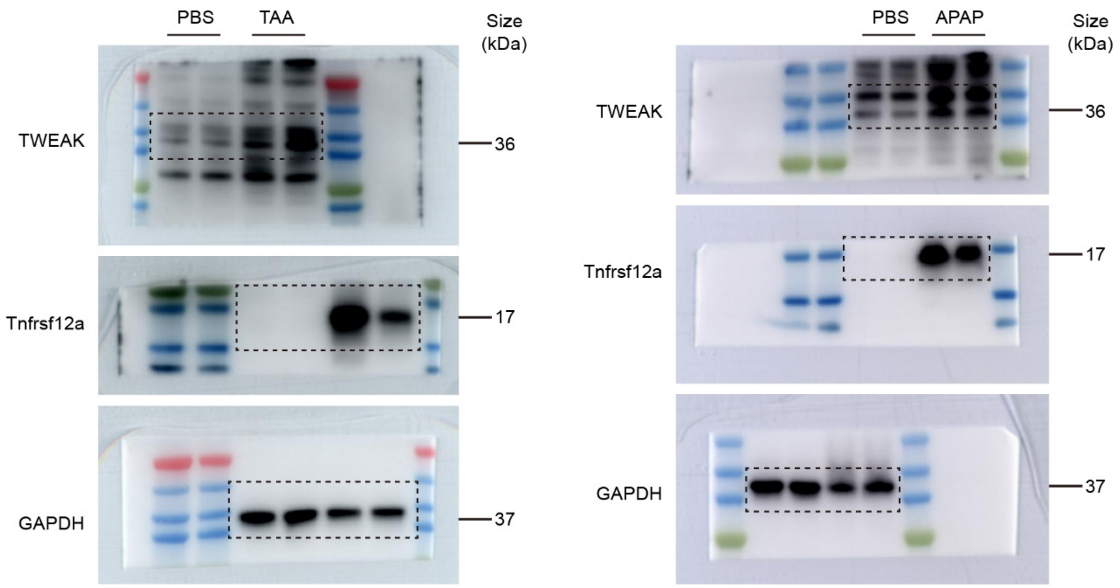

Original image: Figure 3E

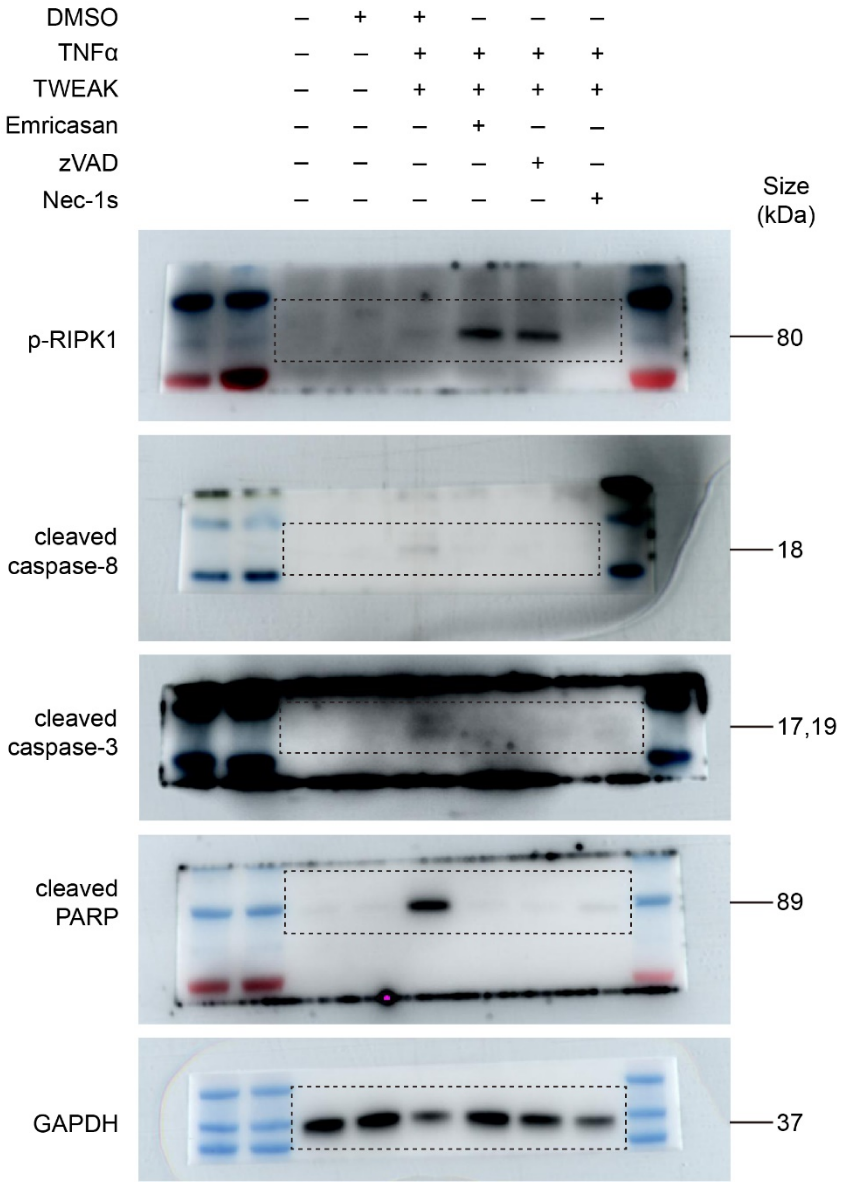

Original image: Figure 3F and 3G

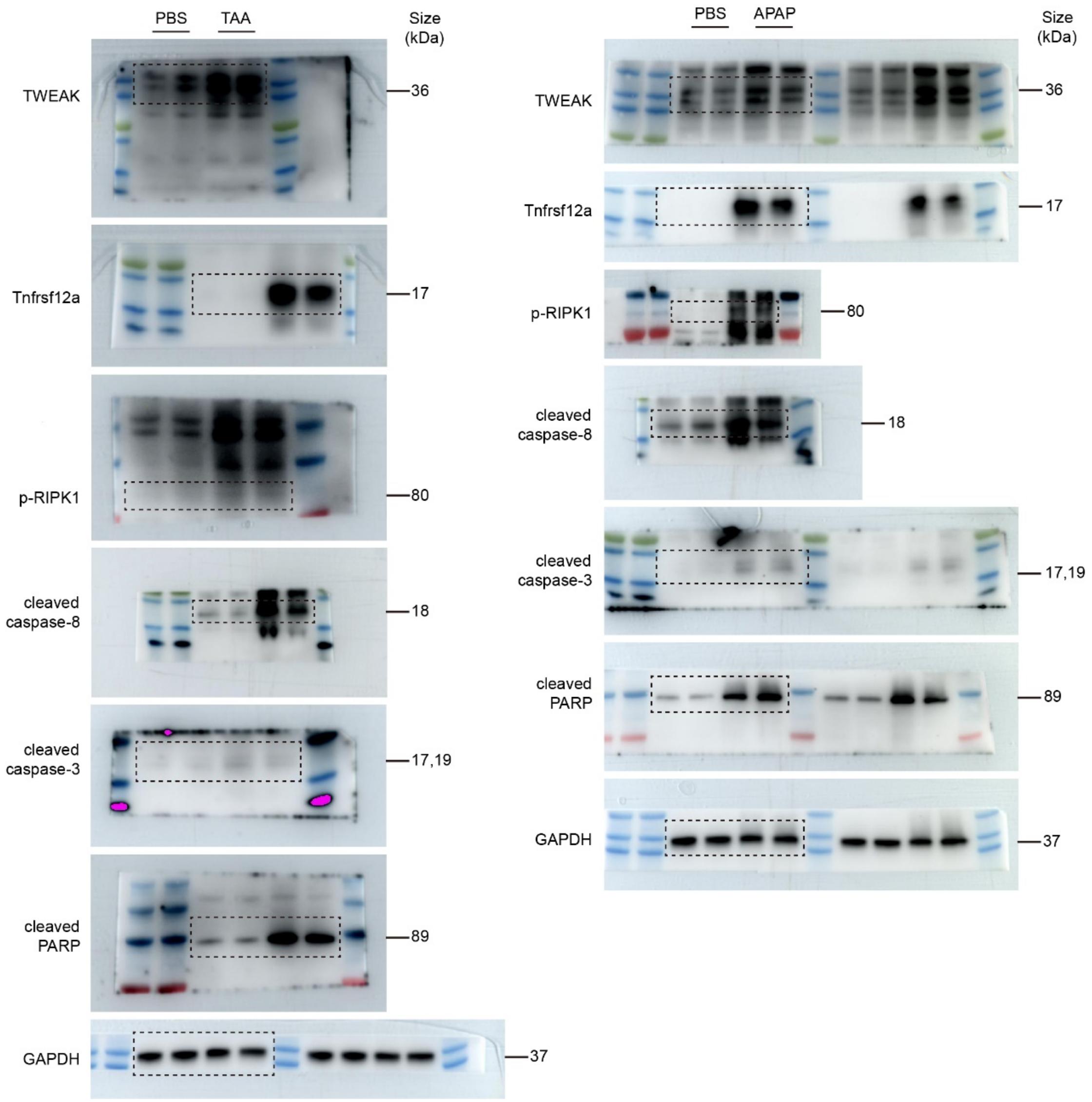

Original image: Figure S7A

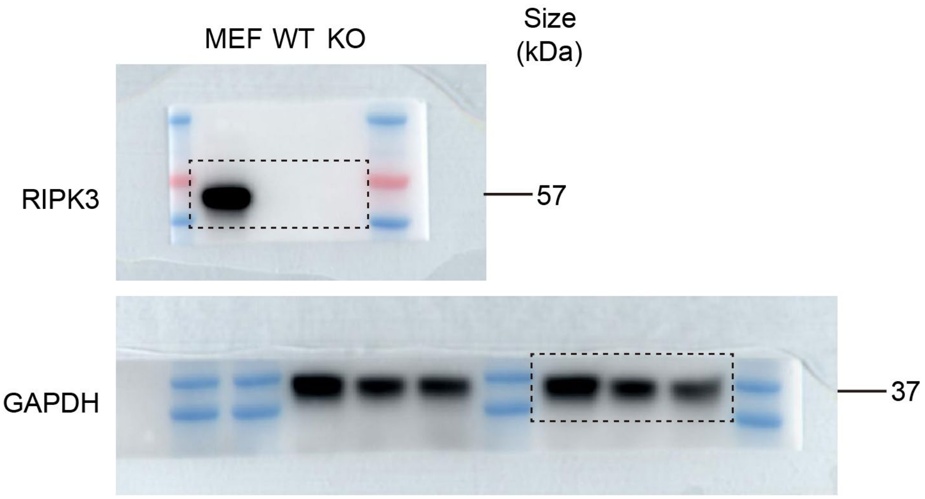

Original image: Figure S7B

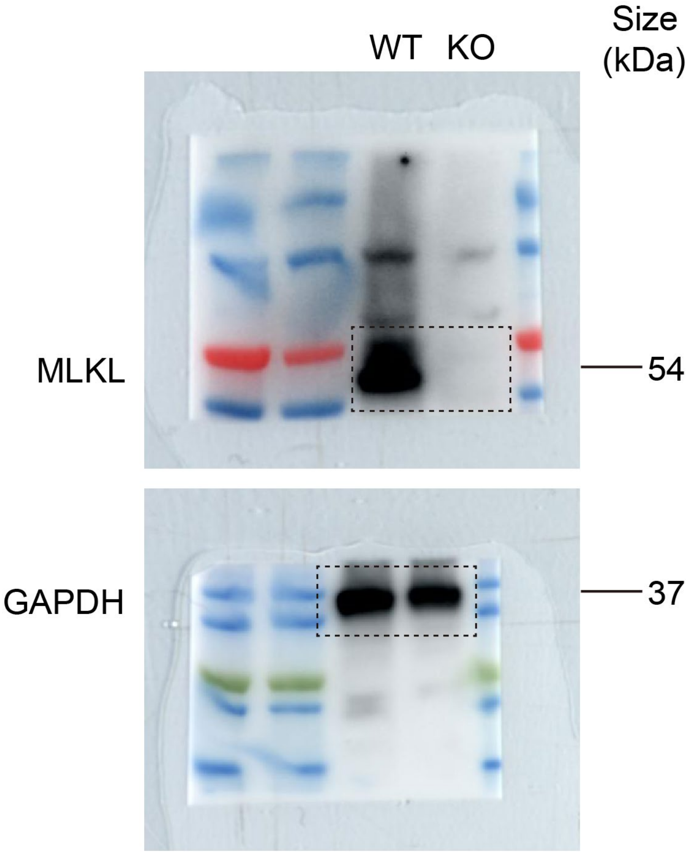

Original image: Figure S7C and S7D

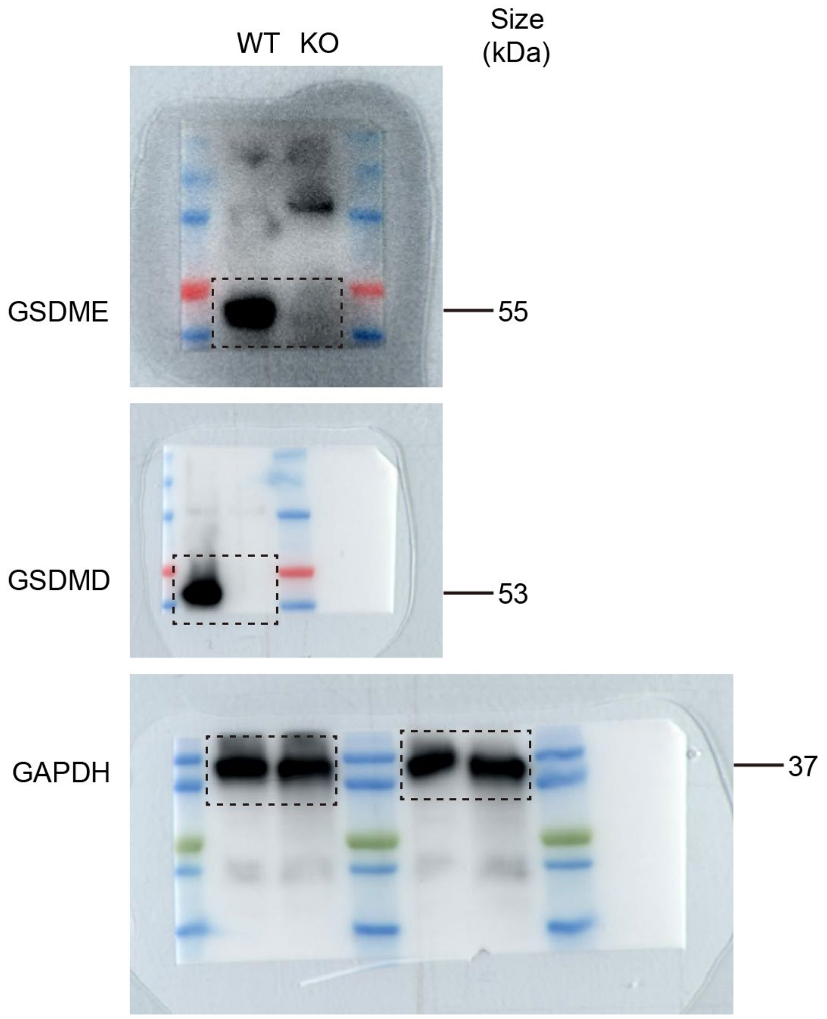

Original image: Figure S7E

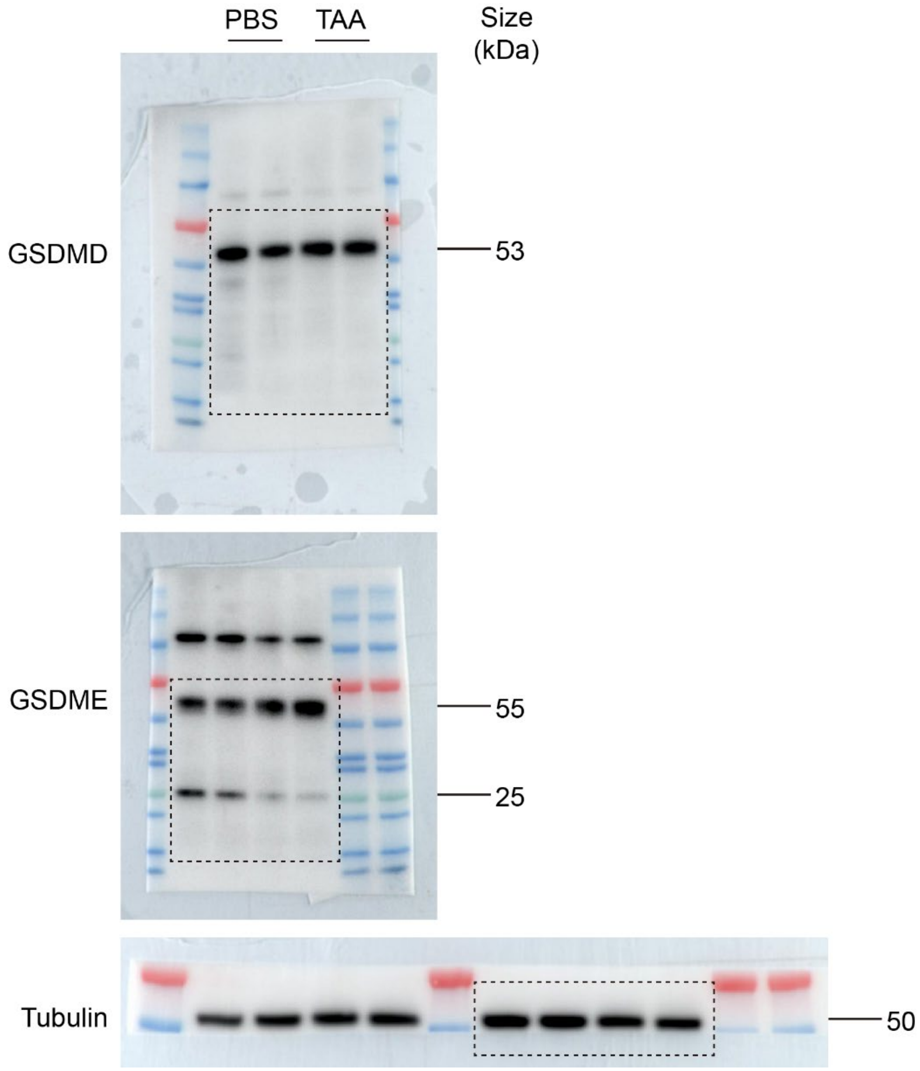

Supplement: Supplementary file 3 — Original Data File [file 41420_2022_1123_MOESM3_ESM.pdf]
