# Supplementary material for: Inhibition of TWEAK/Tnfrsf12a axis protects against acute liver failure by suppressing RIPK1-dependent apoptosis
Source: Cell Death Discov. 2022 Jul 19;8:328. doi: 10.1038/s41420-022-01123-0 (PMC9296540; doi:10.1038/s41420-022-01123-0)
Supplement: Supplementary file 4 — Agreement with the change of authorship [file 41420_2022_1123_MOESM4_ESM.pdf]

Zhijie Li < lizhj@shanghaitech.edu.cn >

答复: Agreement of the change of authorship (Cell Death Discovery) 2022/7/5 14:49

答复: Agreement of the change of authorship (Cell Death Discovery)

"李志杰" <lizhj@shanghaitech.edu.cn>

收件人：huangpengyu <huangpengyu@yeah.net>

时　间：2022-7-5 14:47:54

附　件：

Agree

发件人: huangpengyu [huangpengyu@yeah.net]

发送时间: 2022年7月5日 14:20

收件人: 李志杰; heming_wang; 朱军进; 南宁; 林奕; 庄绪冉; 李玲; 13802122219

主题: Agreement of the change of authorship (Cell Death Discovery)

Dear co-authors,

We have submitted the revised manuscript "Inhibition of TWEAK/Tnfrsf12a axis protects against acute liver failure by suppressing RIPK1-dependent apoptosis" to Cell Death Discovery. During the revision, Dr. Yamin Zhang participated in the supervision of the project and discussion of the manuscript. Therefore, we should add him as one of the corresponding authors. If you agree to the authorship change, please response to the email with "Agree". Thank you.

Yours Sincerely

Pengyu Huang

____

Pengyu Huang, PhD
Deputy Director, Principle Investigator
Institute of Biomedical Engineering
Chinese Academy of Medical Sciences & Peking Union Medical College
Rm 401, No. 2 Bldg, 236 Baidi Road
Nankai District, Tianjin, P.R.China, 300192

Tel: +86-22-87551329

Heming Wang < heming_wang@outlook.com >

答复: Agreement of the change of authorship (Cell Death Discovery) 2022/7/5 15:40

答复: Agreement of the change of authorship (Cell Death Discovery)

"wang heming" <heming_wang@outlook.com>

收件人: huangpengyu <huangpengyu@yeah.net>

时 间: 2022-7-5 15:39:38

附 件:

Agree

发件人: huangpengyu <huangpengyu@yeah.net>
发送时间: 2022年7月5日 14:20
收件人: lizhj <lizhj@shanghaitech.edu.cn>; heming_wang <heming_wang@outlook.com>; zhujj1 <zhujj1@shanghaitech.edu.cn>; nanning <nanning@shanghaitech.edu.cn>; linyi <linyi@shanghaitech.edu.cn>; zhuangxr <zhuangxr@shanghaitech.edu.cn>; liling1 <liling1@shanghaitech.edu.cn>; 13802122219 <13802122219@163.com>
主题: Agreement of the change of authorship (Cell Death Discovery)

Dear co-authors,

We have submitted the revised manuscript "Inhibition of TWEAK/Tnfrsf12a axis protects against acute liver failure by suppressing RIPK1-dependent apoptosis" to Cell Death Discovery. During the revision, Dr. Yamin Zhang participated in the supervision of the project and discussion of the manuscript. Therefore, we should add him as one of the corresponding authors. If you agree to the authorship change, please response to the email with "Agree". Thank you.

Yours Sincerely

Pengyu Huang

———————————

Pengyu Huang, PhD
Deputy Director, Principle Investigator
Institute of Biomedical Engineering
Chinese Academy of Medical Sciences & Peking Union Medical College
Rm 401, No. 2 Bldg, 236 Baidi Road
Nankai District, Tianjin, P.R.China, 300192
Tel: +86-22-87551329

Junjin Zhu < zhujj1@shanghaitech.edu.cn >

Re: Agreement of the change of authorship (Cell Death Discovery) 2022/7/5 15:04

Re: Agreement of the change of authorship (Cell Death Discovery)

zhujj1 <zhujj1@shanghaitech.edu.cn>

收件人：huangpengyu <huangpengyu@yeah.net>

时　间：2022-7-5 15:00:48

附　件：

Agree.

---- Replied Message ----

From huangpengyu<huangpengyu@yeah.net>

Date 07/05/2022 14:20

To lizhj<lizhj@shanghaitech.edu.cn>,
heming_wang<heming_wang@outlook.com>,
zhujj1<zhujj1@shanghaitech.edu.cn>,
nanning<nanning@shanghaitech.edu.cn>,
linyi<linyi@shanghaitech.edu.cn>,
zhuangxr<zhuangxr@shanghaitech.edu.cn>,
liling1<liling1@shanghaitech.edu.cn>,
13802122219<13802122219@163.com>

Subject Agreement of the change of authorship (Cell Death Discovery)

Dear co-authors,

We have submitted the revised manuscript "Inhibition of TWEAK/Tnfrsf12a axis protects against acute liver failure by suppressing RIPK1-dependent apoptosis" to Cell Death Discovery. During the revision, Dr. Yamin Zhang participated in the supervision of the project and discussion of the manuscript. Therefore, we should add him as one of the corresponding authors. If you agree to the authorship change, please response to the email with "Agree". Thank you.

Yours Sincerely

Pengyu Huang

____

Pengyu Huang, PhD
Deputy Director, Principle Investigator
Institute of Biomedical Engineering
Chinese Academy of Medical Sciences & Peking Union Medical College
Rm 401, No. 2 Bldg, 236 Baidi Road
Nankai District, Tianjin, P.R.China, 300192

Tel: +86-22-87551329

Ning Nan < nanning@shanghaitech.edu.cn >

Re: Agreement of the change of authorship (Cell Death Discovery) 2022/7/5 15:04

Re: Agreement of the change of authorship (Cell Death Discovery)

nanning <nanning@shanghaitech.edu.cn>

收件人: huangpengyu <huangpengyu@yeah.net>

时 间: 2022-7-5 15:02:56

附 件:

Agree

发自我的iPhone

------------------ Original ------------------

From: huangpengyu <huangpengyu@yeah.net>
Date: Tue,Jul 5,2022 2:20 PM
To: lizhj <lizhj@shanghaitech.edu.cn>, heming_wang <heming_wang@outlook.com>, zhujj1 <zhujj1@shanghaitech.edu.cn>, nanning <nanning@shanghaitech.edu.cn>, linyi <linyi@shanghaitech.edu.cn>, zhuangxr <zhuangxr@shanghaitech.edu.cn>, liling1 <liling1@shanghaitech.edu.cn>, 13802122219 <13802122219@163.com>
Subject: Re: Agreement of the change of authorship (Cell Death Discovery)

Dear co-authors,

We have submitted the revised manuscript "Inhibition of TWEAK/Tnfrsf12a axis protects against acute liver failure by suppressing RIPK1-dependent apoptosis" to Cell Death Discovery. During the revision, Dr. Yamin Zhang participated in the supervision of the project and discussion of the manuscript. Therefore, we should add him as one of the corresponding authors. If you agree to the authorship change, please response to the email with “Agree”. Thank you.

Yours Sincerely

Pengyu Huang

Pengyu Huang, PhD
Deputy Director, Principle Investigator
Institute of Biomedical Engineering
Chinese Academy of Medical Sciences & Peking Union Medical College
Rm 401, No. 2 Bldg, 236 Baidi Road
Nankai District, Tianjin, P.R.China, 300192

Tel: +86-22-87551329

Yi Lin < linyi@shanghaitech.edu.cn >

Re: Agreement of the change of authorship (Cell Death Discovery) 2022/7/5 14:49

Re: Agreement of the change of authorship (Cell Death Discovery)

"林奕" <linyi@shanghaitech.edu.cn>

收件人: huangpengyu <huangpengyu@yeah.net>

时　间: 2022-7-5 14:46:37

附　件:

Agree

2022年7月5日 14:20，huangpengyu <huangpengyu@yeah.net> 写道:

Dear co-authors,

We have submitted the revised manuscript "Inhibition of TWEAK/Tnfrsf12a axis protects against acute liver failure by suppressing RIPK1-dependent apoptosis" to Cell Death Discovery. During the revision, Dr. Yamin Zhang participated in the supervision of the project and discussion of the manuscript. Therefore, we should add him as one of the corresponding authors. If you agree to the authorship change, please response to the email with "Agree". Thank you.

Yours Sincerely

Pengyu Huang

Pengyu Huang, PhD
Deputy Director, Principle Investigator
Institute of Biomedical Engineering
Chinese Academy of Medical Sciences & Peking Union Medical College
Rm 401, No. 2 Bldg, 236 Baidi Road
Nankai District, Tianjin, P.R.China, 300192

Tel: +86-22-87551329

Xuran Zhuang < zhuangxr@shanghaitech.edu.cn >

答复: Agreement of the change of authorship (Cell Death Discovery) 2022/7/5 14:59

答复: Agreement of the change of authorship (Cell Death Discovery)

"庄绪冉" <zhuangxr@shanghaitech.edu.cn>

收件人: huangpengyu <huangpengyu@yeah.net>

时 间: 2022-7-5 14:51:44

附 件:

Agree

发件人: huangpengyu [huangpengyu@yeah.net]

发送时间: 2022年7月5日 14:20

收件人: 李志杰; heming_wang; 朱军进; 南宁; 林奕; 庄绪冉; 李玲; 13802122219

主题: Agreement of the change of authorship (Cell Death Discovery)

Dear co-authors,

We have submitted the revised manuscript "Inhibition of TWEAK/Tnfrsf12a axis protects against acute liver failure by suppressing RIPK1-dependent apoptosis" to Cell Death Discovery. During the revision, Dr. Yamin Zhang participated in the supervision of the project and discussion of the manuscript. Therefore, we should add him as one of the corresponding authors. If you agree to the authorship change, please response to the email with "Agree". Thank you.

Yours Sincerely

Pengyu Huang

———————

Pengyu Huang, PhD
Deputy Director, Principle Investigator
Institute of Biomedical Engineering
Chinese Academy of Medical Sciences & Peking Union Medical College
Rm 401, No. 2 Bldg, 236 Baidi Road
Nankai District, Tianjin, P.R.China, 300192

Tel: +86-22-87551329

Ling Li < liling1@shanghaitech.edu.cn >

答复: Agreement of the change of authorship (Cell Death Discovery) 2022/7/5 15:40

答复: Agreement of the change of authorship (Cell Death Discovery)

"李玲" <liling1@shanghaitech.edu.cn>

收件人: huangpengyu <huangpengyu@yeah.net>

时　间: 2022-7-5 15:36:56

附　件:

Agree!

发件人: huangpengyu [huangpengyu@yeah.net]

发送时间: 2022年7月5日 14:20

收件人: 李志杰; heming_wang; 朱军进; 南宁; 林奕; 庄绪冉; 李玲; 13802122219

主题: Agreement of the change of authorship (Cell Death Discovery)

Dear co-authors,

We have submitted the revised manuscript "Inhibition of TWEAK/Tnfrsf12a axis protects against acute liver failure by suppressing RIPK1-dependent apoptosis” to Cell Death Discovery. During the revision, Dr. Yamin Zhang participated in the supervision of the project and discussion of the manuscript. Therefore, we should add him as one of the corresponding authors. If you agree to the authorship change, please response to the email with “Agree”. Thank you.

Yours Sincerely

Pengyu Huang

____

Pengyu Huang, PhD
Deputy Director, Principle Investigator
Institute of Biomedical Engineering
Chinese Academy of Medical Sciences & Peking Union Medical College
Rm 401, No. 2 Bldg, 236 Baidi Road
Nankai District, Tianjin, P.R.China, 300192

Tel: +86-22-87551329

Yamin Zhang < 13802122219@163.com >

Re:Agreement of the change of authorship (Cell Death Discovery) 2022/7/5 16:10

Re:Agreement of the change of authorship (Cell Death Discovery)

"张雅敏" <13802122219@163.com>

收件人: huangpengyu <huangpengyu@yeah.net>

时 间: 2022-7-5 16:02:53

附 件:

Agree

At 2022-07-05 14:20:29, "huangpengyu" <huangpengyu@yeah.net> wrote:

> Dear co-authors,
>
> We have submitted the revised manuscript "Inhibition of TWEAK/Tnfrsf12a axis protects against acute liver failure by suppressing RIPK1-dependent apoptosis" to Cell Death Discovery. During the revision, Dr. Yamin Zhang participated in the supervision of the project and discussion of the manuscript. Therefore, we should add him as one of the corresponding authors. If you agree to the authorship change, please response to the email with "Agree". Thank you.
>
> Yours Sincerely
>
> Pengyu Huang
>
> ________________
>
> Pengyu Huang, PhD
> Deputy Director, Principle Investigator
> Institute of Biomedical Engineering
> Chinese Academy of Medical Sciences & Peking Union Medical College
> Rm 401, No. 2 Bldg, 236 Baidi Road
> Nankai District, Tianjin, P.R.China, 300192
>
> Tel: +86-22-87551329
